# *In Situ* Observation of Chemically Induced Protein Denaturation at Solvated Interfaces

**DOI:** 10.1021/acsami.3c10510

**Published:** 2023-10-05

**Authors:** Peter Niraj Nirmalraj, Marta D. Rossell, Walid Dachraoui, Damien Thompson, Michael Mayer

**Affiliations:** † Transport at Nanoscale Interfaces Laboratory, Swiss Federal Laboratories for Materials Science and Technology, Überlandstrasse 129, 8600 Dübendorf, Switzerland; ‡ Electron Microscopy Center, Swiss Federal Laboratories for Materials Science and Technology, Überlandstrasse 129, 8600 Dübendorf, Switzerland; § Department of Physics, Bernal Institute, University of Limerick, Limerick V94T9PX, Ireland; ∥ Adolphe Merkle Institute, University of Fribourg, Chemin des Verdiers 4, CH-1700 Fribourg, Switzerland

**Keywords:** protein unfolding, ferritin, nanoring, graphene, atomic force microscopy, molecular dynamics simulations

## Abstract

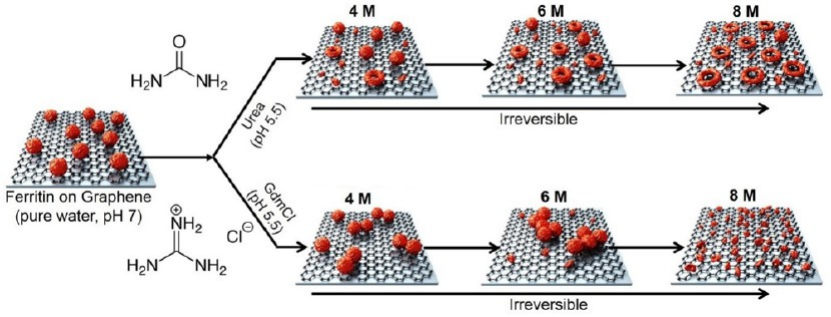

Proteins unfold in chaotropic salt solutions, a process that is difficult to observe at the single protein level. The work presented here demonstrates that a liquid-based atomic force microscope and graphene liquid-cell-based scanning transmission electron microscope make it possible to observe chemically induced protein unfolding. To illustrate this capability, ferritin proteins were deposited on a graphene surface, and the concentration-dependent urea- or guanidinium-induced changes of morphology were monitored for holo-ferritin with its ferrihydrite core as well as apo-ferritin without this core. Depending on the chaotropic agent the liquid-based imaging setup captured an unexpected transformation of natively folded holo-ferritin proteins into rings after urea treatment but not after guanidinium treatment. Urea treatment of apo-ferritin did not result in nanorings, confirming that nanorings are a specific signature of denaturation of holo-ferritins after exposture to sufficiently high urea concentrations. Mapping the *in situ* images with molecular dynamics simulations of ferritin subunits in urea solutions suggests that electrostatic destabilization triggers denaturation of ferritin as urea makes direct contact with the protein and also disrupts the water H-bonding network in the ferritin solvation shell. Our findings deepen the understanding of protein denaturation studied using label-free techniques operating at the solid–liquid interface.

## Introduction

The ability to predict how the structure of proteins and nucleic acids changes in response to physical and chemical stresses is essential to prevent biomolecular denaturation in applications ranging from point-of-care diagnostics to drug encapsulation and delivery to cells. External stimuli such as heat,^1^ high pressure,^2^ low temperature,^3^ chemicals,^4−6^ radiation,^7^ ultrasound,^8^ and interactions with surfaces can alter the native conformation and function of proteins. In some cases, unfolded proteins can retain the ability to refold and regain function,^9^ upon removal of the stimulus that triggered denaturation.

The use of chaotropic agents as a means to shift the conformation of native proteins is a century-old field of study in biochemistry.^6^ Notably, in 1972 the work of Christian Anfinsen on protein refolding after denaturation received the Nobel Prize in chemistry. Since then there has been a quest to study the effect of these chaotropic agents on natively folded proteins using circular dichroism (CD),^5^ small-angle X-ray scattering,^10^ nuclear magnetic resonance spectroscopy (NMR),^11^ electron microscopy,^5^ and single-molecule fluorescence studies.^12^ More recently molecular dynamics (MD) computer simulations of protein denaturation have complemented experiments by revealing direct and water-mediated interactions of urea with lysozyme,^13^ identifying the chemical denaturation process of chymotrypsin by urea^14^ and elucidating the effect of urea on water structure.^15^

Traditionally, urea and guanidinium chloride (GdmCl) have been chiefly used as the chemical denaturant of choice in protein denaturation studies owing to their ability to selectively bind to peptide groups^16^ and screen intramolecular hydrogen bonds and affect the tertiary and secondary structure but not the primary structure of the proteins. Despite significant advances in the experimental and simulation-guided understanding of the chemical denaturation of proteins,^4−6,15−27^ there are only a limited number of studies that directly visualize the morphology of the partially unfolded and denatured states of proteins. While early electron microscopy studies^5^ indicated GdmCl-driven denaturation of ferritin proteins, the technique still required treating the proteins with uranyl acetate (negative staining) and air-drying before imaging, which could affect the denaturation of the proteins,^25^ thereby making it difficult to isolate the effect of GdmCl. An alternative *in situ* technique that remains to be fully exploited for studying protein denaturation is liquid-based atomic force microscopy (AFM) in a sample chamber with an aqueous liquid. Although several reports on the application of liquid-based AFM provide spectacular details on single-protein folding and unfolding events in aqueous solutions,^28,29^ the approach to the best of our knowledge has not previously been used to image the size and shape changes of individual nanometer-sized proteins in a medium with chaotropic agents in solutions. One of the main factors hindering the reliable operation of an AFM probe in urea and GdmCl solutions is the contamination of the probe apex by the encompassing salts or protein subunits present in chaotropic solutions, which can lead to an erroneous recording of actual protein morphology. Changes in protein conformation during adsorption on surfaces^30,31^ and self-assembly and aggregation^32^ have been resolved in detail, albeit not in real space in the presence of chemical denaturants.

Here, we provide direct visualization by AFM of the effect of urea and GdmCl on the shape of ferritins at the single-protein level on epitaxially grown graphene on silicon carbide (SiC). We demonstrate that a carefully prepared AFM tip made from diamond-like carbon (DLC) with an aluminum reflex coating (see the Supporting Information Section 1 for details on tip specification, storage, cleaning, and AFM operation procedure) makes it possible to image the morphological changes of ferritin proteins in both urea and GdmCl. After screening several other AFM probes (solid metal, metal coated, and SiN_3_), we identified the DLC AFM tip to be the most resistant against urea and GdmCl-induced tip surface corrosion and contamination. Using this probe in tapping mode, we were able to distinguish between natively folded and non-native states of ferritin proteins deposited on epitaxially grown graphene on SiC. Ferritin proteins are large 24-unit iron storage proteins with a diameter of ∼12 nm.^33^ We chose to investigate ferritin proteins because of their characteristic shape in the native state, which is well-known from nanoscale imaging studies.^5,34−36^

We investigated the concentration-dependent unfolding effects on ferritin proteins of both chemical denaturants (at 4, 6, and 8 M, with pH 5.5). Upon exposure to urea at all three concentrations, holo-ferritin unfolded into nanoscale rings with a height between 2 and 8 nm and an inner cavity spacing between 2 and 50 nm, as observed from high-resolution AFM images. In contrast to urea, 2, 4, and 6 M GdmCl caused the aggregation of ferritin proteins, while 8 M GdmCl disintegrated ferritins into subunits, confirming that GdmCl is a harsher denaturant than urea with a different mechanism. No ring-type structures were observed at any stage of ferritin denaturation using GdmCl at 4, 6, and 8 M concentrations, suggesting that the observed ring formation is a urea-specific effect. As a further control, we confirmed that apoferritin proteins, which do not contain the ferrihydrite core, exposed to urea, did not show any ring-type structures. Reimaging the ferritin proteins on graphene after removal of urea and GdmCl by gentle liquid exchange with pure water did not show any evidence of refolding, suggesting that urea and GdmCl have an irreversible denaturation effect on graphene-associated ferritin proteins. Additional experiments were also performed using scanning transmission electron microscopy (STEM) to study the effect of 6 M urea on ferritin proteins within graphene liquid cells (GLC), which confirmed nanoring formation, thus independently validating the results obtained from AFM. Molecular dynamics computer simulations show how ferritin is engaging in large-area van der Waals interactions with the graphene surface in water and show the atomic-scale destabilization of the ferritin peptide monomer in 8 M urea. We provide a detailed qualitative and quantitative analysis of the size distributions of ferritin conformations upon exposure to urea and GdmCl. We proposed that the liquid AFM and STEM *in situ* imaging details presented here can be applied in the future to study the nanoscopic changes that biomolecules, from DNA origami structures^37^ to proteins,^38^ undergo when treated with chemicals.

## Results and Discussion

### Profiling Natively Folded Ferritin Proteins at Solvated Interfaces

**Figure 1:**
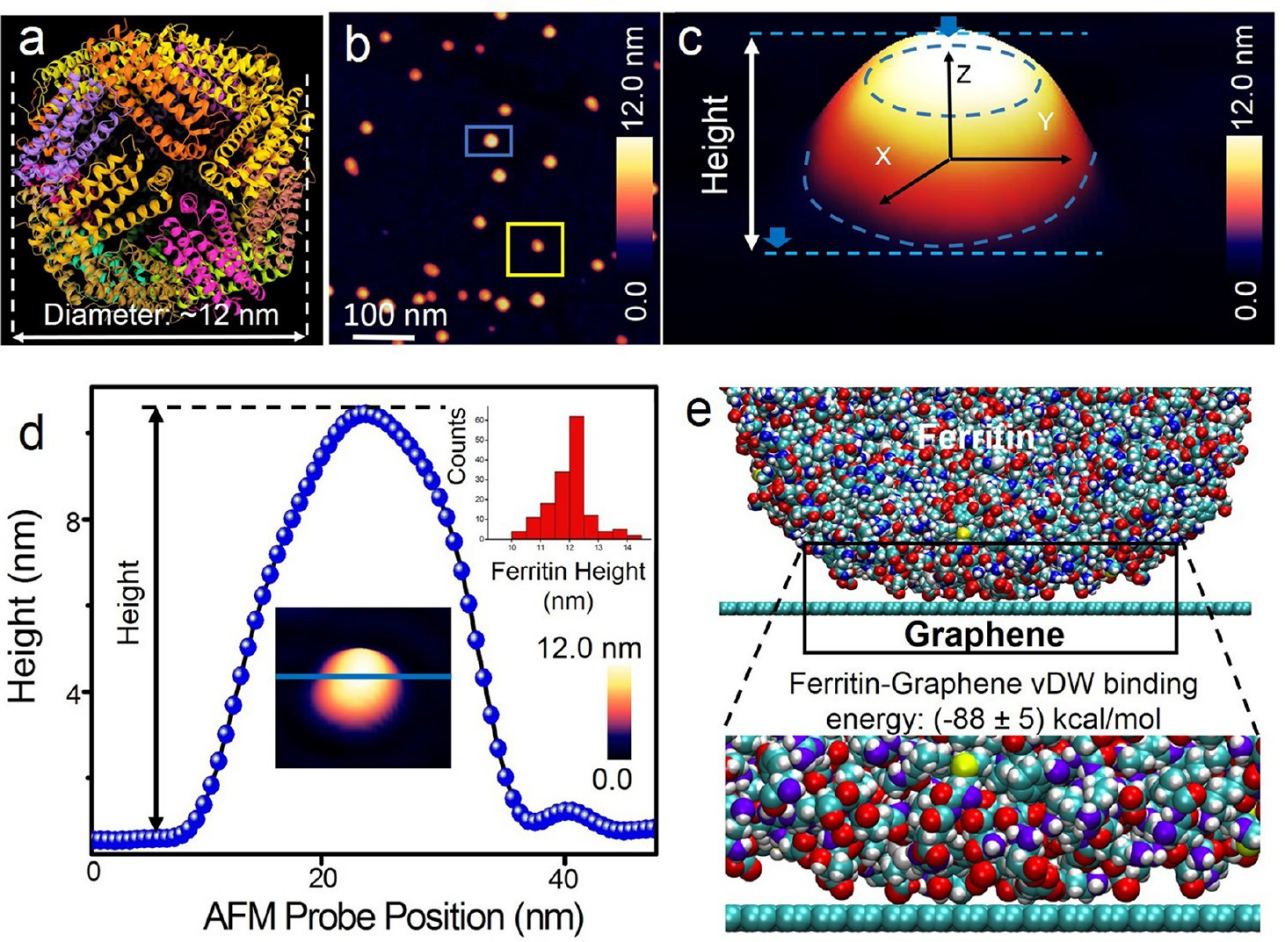
Imaging natively folded ferritin proteins at the graphene–liquid interface. (a) Cryo-electron microscopy structure of human ferritin at 3.0 Å resolution (PDB identifier: 5XB1(39)). (b) Large-area AFM topographic image of isolated ferritin proteins on graphene with varying height as shown in example particles indicated by a blue and yellow box. (c) Spatially magnified and three-dimensionally represented AFM height image of ferritin (indicated within the blue box in panel b) with globular morphology. (d) AFM sectional profile (extracted along the blue line in the inset AFM image) reveals the measured height and convoluted (AFM tip–sample convolution) diameter of a single ferritin protein measured at a different region of the same sample. The top right inset in the panel is the statistical analysis of ferritin height obtained from the cross-sectional analysis of single ferritin proteins from AFM data. (e) Snapshot from molecular dynamics simulation of the interactions of ferritin protein on the graphene surface. Future details on computation models, methods, and analyses are given in Supporting Information Section S3.

Figure 1a shows a 3.0 Å resolution structure of human ferritin proteins obtained using cryo-electron microscopy (protein data bank identifier: 5Y15(39)). The globular structure of ferritin (molecular weight: ∼450 kDa) is composed of 24 subunits (H- and L-chains) with an inner cavity for iron storage known from previous studies.^40−42^ Here, we used L-chain-rich ferritins isolated from the human liver with their ferrihydrite core (see the Supporting Information Section S1 for details on sample preparation and buffer conditions). The large-area AFM scan in Figure 1b shows single ferritin proteins randomly adsorbed at the graphene water interface. We deposited ferritin proteins first on a freshly cleaned graphene surface from a buffered aqueous solution (150 mM NaCl, 10 mM Tris, pH 8.0, and 0.1% sodium azide), and after ∼2 min, the graphene surface was flushed with pure water to remove adsorbed residues from the buffer solution. The rationale for first imaging natively folded ferritins with a DLC AFM tip in water was to measure and benchmark their size, shape, and height in a clean environment before exposing the ferritin proteins to urea and GdmCl. From the AFM measurements, we did not observe lateral diffusion of the adsorbed ferritin proteins on the graphene surface, which is an added benefit for stable AFM imaging. Figure 1c is a high-resolution 3D reconstructed AFM image of single ferritin (marked by the blue box in the large-area AFM scan in Figure 1b). The semispherical shape and *z*-height of the globular protein as a result of adsorption on a solid surface are visible from the 3-D AFM topography. A cross-sectional profile measured across a single ferritin protein (Figure 1d) resolved in a different region of the same sample provides quantitative information on the height and particle diameter. The height of ∼12 nm suggests that the ferritins adsorbed on the graphene surface are not structurally denatured, hence excluding surface-induced morphological changes of the proteins. This aspect of surface effects on the size and shape of the adsorbed proteins needs to be recorded before intentionally inducing conformational changes through an intentionally applied stimulus such as heat, chemical forces, or mechanical forces to be able to study the specific effects of the external stimulus on the surface-confined proteins.

Based on sectional analyses on 143 single ferritin proteins, we calculate a mean ferritin height of 11.6 ± 0.7 nm (statistical analysis is shown in the top right inset of Figure 1d) for ferritin adsorbed at the graphene–water interface, which is consistent with previous AFM reports^35,43^ on ferritin morphology. The apparent diameter of the single ferritin from the cross-sectional profile is almost twice the value of the actual diameter of ferritin (∼12 nm). This increased diameter is a result of convolution between the DLC AFM tip (apex radius: ∼10 nm) and the globular ferritin protein. Because of this tip-induced broadening effect, the diameter of ferritin cannot be accurately determined. Nonetheless, the height of ferritin protein adsorbed on graphene is not influenced by the geometry of the AFM tip, and the height equals the diameter of a globular protein with an almost perfectly spherical shape, such as ferritins. This property makes it possible to estimate the size of the ferritin proteins from the height values obtained from the AFM topographs. By using an ultrasharp AFM tip (apex radius: <2 nm), it should be possible to get a closer diameter match to the ferritin crystal structure when measured in pure water. However, the goal of the current study was to operate an AFM tip that is resilient to contamination (corrosion of the tip-apex coating) from solutions containing high-concentration chaotropic agents and to obtain direct visual evidence of proteins in various stages of unfolding in such solutions. For this purpose, we identified that a DLC coating (which then increases the Si tip apex radius) is essential for providing consistent images of the unfolded states of ferritin while remaining uncontaminated in urea and GdmCl solution for prolonged periods of 60–90 min after which the tip required cleaning (see Supporting Information Section 2 for DLC tip cleaning procedure after exposure to urea and GdmCl).

To gain deeper insights into the ferritin–graphene interface, MD simulations were used to quantify the interfacial energies. Figure 1e is a typical computed structure of the ferritin graphene interface during 100 ns of free room temperature dynamics in water from which we calculated a time-averaged van der Waals binding energy of −88 ± 5 kcal/mol, sampling every 10 ps during the final 50 ns of dynamics). On average, 9 ± 1 residues of the adsorbed ferritin protein subunit make contact with the graphene surface, creating binding energies of approximately −10 kcal/mol or 430 meV each, with negligible conformational penalties in the protein. The MD data on the interaction energies between ferritin and graphene show reversible interactions, consistent with the previous MD studies of large globular proteins on carbon surfaces.^44^ Taken together with the shape and height profile information from the natively folded ferritin proteins from the AFM measurements, these findings suggest that graphene could potentially be a reliable platform to image the native structure of single proteins.

### Studying Urea Effects on Holo-Ferritins Using Liquid-AFM and Simulations

**Figure 2:**
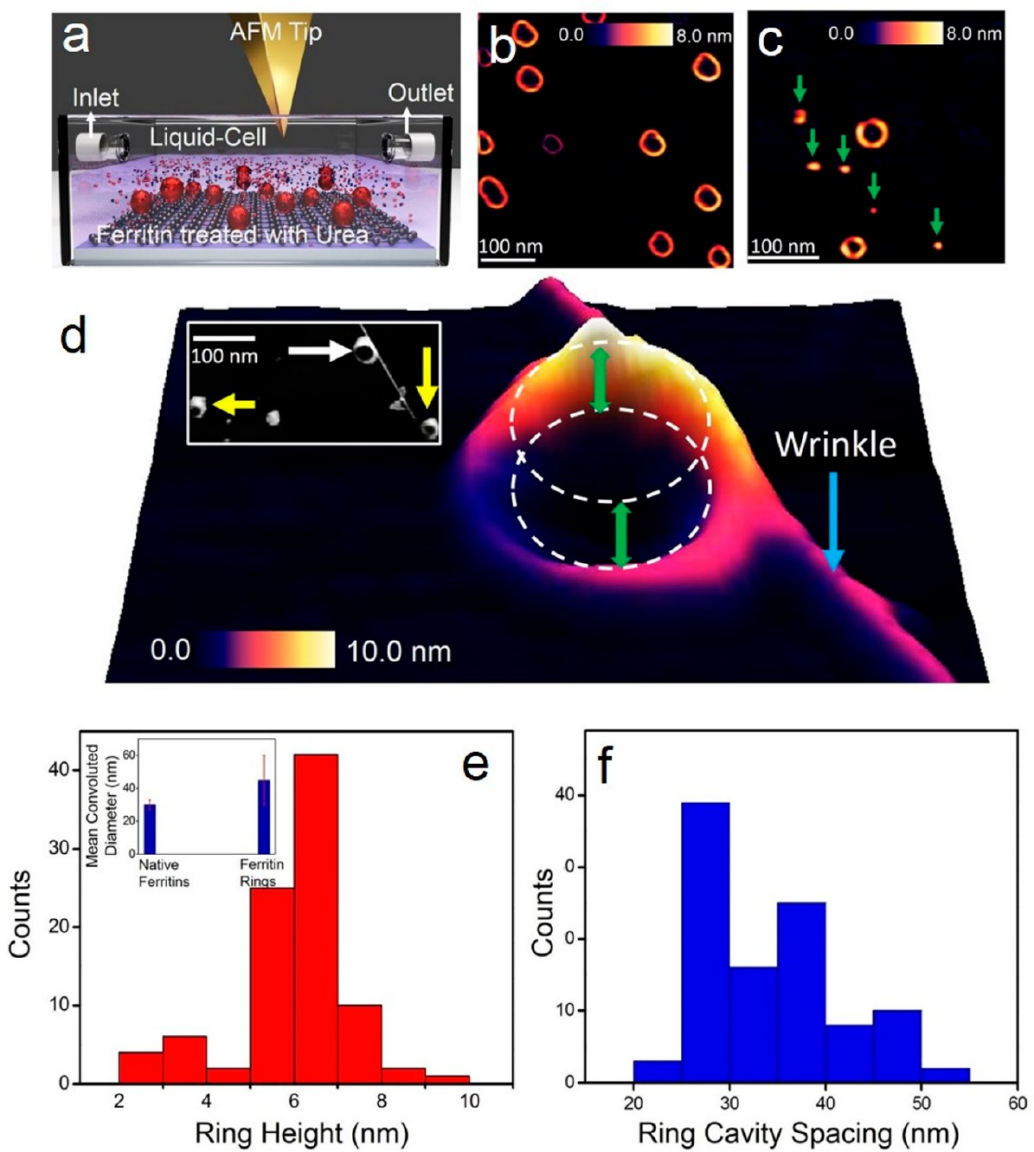
Resolving urea effects on holo-ferritins adsorbed on graphene (a) Schematic of liquid-AFM setup for studying urea effects on ferritins adsorbed on graphene. (b) Large-area AFM image shows the presence of ring-structured ferritins with different height and size profiles when natively folded ferritin proteins were first adsorbed on graphene and subsequently exposed to 8 M urea salt solution. (c) AFM image revealing the presence of ferritin nanorings and smaller units (indicated by green arrows). (d) 3D-rendered AFM image of a ferritin ring resolved in the vicinity of an out-of-plane wrinkle (indicated by the blue arrow). Local differences in the height of the denatured ferritin ring upon urea exposure are visible from the 3-D AFM topography. Inset in panel d is a large-area AFM image (in grayscale) showing more ferritin rings and intact ferritin. (e) Statistical analysis of nanoring height of 5.7 ± 1.2 nm. The height profile of a single ferritin ring is calculated based on the difference in height between the edge of the ring and the underlying surface. The other inset (top left) in the panel is a plot of the mean convoluted diameter of native ferritin protein and denatured ferritin rings, with a larger diameter measured for the ferritin rings in comparison to the native globular-shaped ferritins. (f) The statistical plot provides a mean cavity spacing value of 31.5 ± 7.3 nm (includes tip-sample convolution effects) for ferritin proteins treated with 8 M urea.

For the chemically induced protein unfolding experiments, we first investigated the influence of urea (8 M, pH 5.5) on individual holo-ferritin proteins adsorbed on graphene placed within the perfusion-type liquid-cell setup, as shown in the schematic (Figure 2a). The rationale for selecting a pH of 5.5 for all concentrations of urea and GdmCl in the current study is that it matches the isoelectric point of ferritins (5.5) isolated from the human liver.^45^ Circular dichroism and gel electrophoresis studies^5^ of the effect of GdmCl and urea on ferritins isolated from horse spleen indicated that the transitions between native and denatured states are most pronounced at a pH of 4.5. Upon confirming through AFM imaging the presence of structurally intact ferritins on graphene in buffer medium, 10 μL of urea (8 M) was injected through the inlet port of the liquid cell (see Supporting Information Section 2 for details on the protocols followed for urea solution preparation and the routine followed for AFM imaging in urea). Typically, morphological changes of ferritin proteins could be observed approximately 1 min after urea exposure due to the requirements of the tip tuning procedure and the time required to establish stable and reproducible imaging. A large-area AFM image recorded with a high spatial resolution (2048 × 2048 pixels, scan rate of 0.5 Hz) reveals the rapid effect of urea on ferritin proteins (Figure 2b) as observed from the presence of ring-like ferritin structures on a graphene surface. This result was surprising as we had initially anticipated that exposing ferritin proteins to urea (8 M) would partially denature the protein and likely result in dissociation into subunits, as chemical denaturants are known to have location-specific interactions in a peptide structure.^46,47^ Note that ferritin proteins deposited on a gold surface and then treated with urea (8 M, pH 5.5) also resulted in nanoscale toroidal rings, indicating that the transformation is not due to specific interactions with graphene (Figure 2b–d). Graphene is our imaging platform of choice for these studies mainly because it offers a more contaminant-free interface for AFM imaging compared with the gold surface. Gold showed a higher tendency to adsorb salts from the chaotropic solution, thus hindering the recording of clean AFM measurements. A large-area scan of ferritin nanorings formed on the Au(111) surface is provided in Figure S3a, and a high-resolution image of a nanoring on a gold surface is shown in Figure S3b.

The *in situ* observation of ferritin protein denaturation into peculiar ring-like structures raised several questions. How prevalent are these structures? Do other denatured states coexist? What is the mechanism behind the formation of the rings? Can the denaturation be reversed? Can ring formation occur at a lower urea concentration? Can a similar effect be reproduced upon exposure of ferritin proteins to GdmCl? Can similar ring structures be formed from apoferritin (without a ferrihydrite core)? Is ring formation partially influenced by the AFM probe? The present work addresses these questions through a combination of experimental (liquid-AFM, liquid-TEM) and MD simulations.

Although the AFM data shown in Figure 2b show predominantly nanoscale rings of unfolded ferritin proteins, we also detected a mixed population of rings, natively folded ferritin proteins, and protein subunits on the graphene surface. Shifting the AFM tip to other locations on the same sample revealed ferritin rings coexisting with smaller units (indicated by green arrows), as shown in Figure 2c. Hence, a clear result from the AFM measurements was the formation of ferritin nanorings, together with smaller subunits and a small population of natively folded ferritin proteins. Figure 2d is a 3-D rendered AFM topograph of a well-resolved ferritin ring adsorbed at the edge of an out-of-plane wrinkle (indicated by the blue arrow in Figure 2d) typically observed on graphene surfaces.^48,49^ Closer inspection of the 3D-projected ferritin ring in Figure 2d reveals the differences in height along the rim and confirms that the ferritin ring itself is hollow in the center. A large-area AFM image in the inset of Figure 2d shows the occurrence of other ring-like structures in the vicinity of the ferritin ring and the presence of smaller-sized ferritin subunits. Based on sectional analysis of the individual ferritin rings, we calculated a mean height of the nanorings in 8 M urea of 5.7 ± 1.2 nm (red bar histogram, Figure 2e).

However, the convoluted diameter (including tip–sample convolution effects) of the ferritin rings is visibly 2-fold larger than natively unfolded ferritin proteins in the AFM images (Figure 2b–d). The deconvolution profile of individual proteins can also be obtained by knowing the exact geometry of the tip.^50^ An additional geometry parameter of the rings that can be obtained from the AFM images is the inner cavity spacing of the hollow rings (the diameter of the hollow part at the center of the toroidal ring). Figure 2f is a plot of the cavity spacing for ferritin proteins denatured in 8 M urea, revealing a mean cavity spacing of 31.8 ± 7.3 nm. In addition to quantifying the geometry of the persistent nanoring structures, we also calculated the size distribution of the dissociated subunit population determined from the AFM data. Figure S4 shows the distribution of the subunit population detected when natively folded ferritin proteins were exposed to 8 M urea. Based on the height profile analysis from the AFM data sets, the mean subunit particle height is 2.0 ± 1.1 nm, which roughly corresponds to the width of a single ferritin subunit.^41^

**Figure 3:**
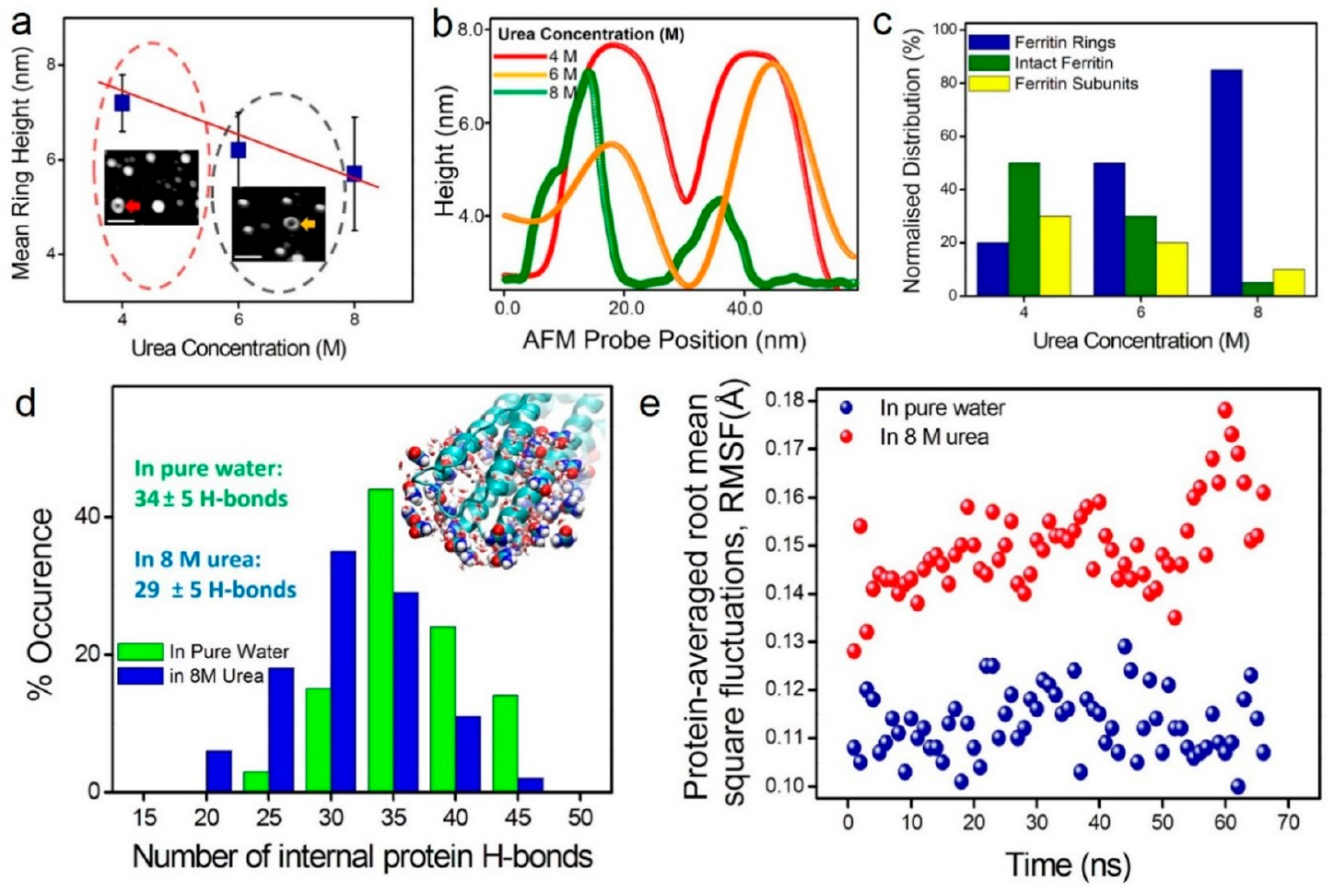
Quantifying differences in ferritin morphology as a function of urea concentration. (a) A plot of mean ferritin ring height as a function of urea concentration. The mean ferritin ring height decreases gradually with increasing concentration of urea. The insets in panel a are AFM images of ferritins proteins exposed to 4 M urea (marked with a red dash oval, the scale bar in the AFM image is 50 nm) and 6 M urea (marked with a black dash oval, the scale bar in the AFM image is 50 nm). (b) Height profiles extracted along individual ferritin rings formed as a result of exposure to native ferritin to 4 M urea (red trace measured across the ferritin ring indicated by the red arrow in panel a), 6 M urea (orange trace measured across the ferritin ring indicated by orange arrow in panel a), and 8 M urea (green trace measured across the ferritin ring indicated by the white arrow in the inset of Figure 1a). (c) Shape distribution of ferritin-derived structures over 100 μm^2^ of the graphene surface. (d) Computed populations of internal H-bonds in ferritin subunit computed over 66 ns of free molecular dynamics showing decreased stability in 8 M urea compared to water. Inset in panel d is a cross-sectional view of a ferritin subunit taken during molecular dynamics in 8 M urea, with protein shown in cartoon representation, protein-coordinating water shown as sticks, and protein-coordinating urea molecules highlighted as vdW space-filling spheres. (e) Plot of residue-averaged root-mean-square fluctuations of a ferritin subunit during 66 ns of molecular dynamics.

Next, we investigated the effect of 4 and 6 M urea concentrations on ferritin proteins and characterized the distribution and size of all ferritin nanorings based on a statistical analysis of the AFM images. The presence of ferritin nanorings is visible from the AFM images recorded in both 4 M (inset in Figure 3a indicated by a red dashed oval) and 6 M (inset in Figure 3a indicated by a black dashed oval) urea solutions. The AFM images under these conditions also indicate the increased abundance of natively folded ferritin proteins and smaller particles, which are presumably individual ferritin subunits, compared to ferritin exposed to 8 M urea. The size distribution (derived from the height profile analysis) indicates a mixture of dissociated ferritin subunits and subunit aggregates. Importantly, we did not observe any ferritin rings at lower urea concentrations of 2 and 3 M, indicating a minimum urea concentration of 4 M was required to trigger the transition of ferritin protein to rings. Figure 3a shows a gradual decrease in the mean height of ferritin nanorings from 7.2 ± 0.6 nm at 4 M urea to 6.2 ± 0.8 nm at 6 M urea and 5.7 ± 1.2 nm at 8 M urea. A closer examination of the AFM data on the ferritin nanorings formed at 4 M urea (Figure 3a, inset), 6 M urea ( Figure 3a, inset), and 8 M urea (Figure 2b–d) shows that the ring structure starts with a small central pore at 4 M urea, which then increases at 6 M urea and finally forms a widely spaced doughnut-shaped ring at 8 M urea solutions. Based on sectional profile analysis along with analysis of diameters from several individual ferritin rings (single traces are shown in Figure 3b) observed in 4 and 6 M urea, we plot the mean height of ferritin nanorings as a function of urea concentration. The tip convoluted inner cavity spacing within the nanorings can be observed from the AFM images (Figure 2b–d and inset in Figure 3a) to swell with an increased concentration of urea. A plot of mean cavity spacing as a function of urea concentration is provided in Figure S5 calculated from sectional profiles as shown in Figure 2b.

Figure 3c is an overview of the distribution (obtained over 100 μm^2^ on several graphene samples) of ferritin nanorings, natively folded ferritin proteins, and ferritin subunits observed from the AFM images recorded when native ferritin proteins adsorbed on graphene were exposed to 4, 6, and 8 M urea. A larger population of ferritin rings was observed at 8 M urea concentration, together with a lower occurrence of natively folded ferritin and its subunits compared with the distribution at 4 and 6 M urea concentrations. A plausible explanation for this trend in the distribution profile of unfolded ferritins is that at higher urea concentrations, the natively folded ferritin proteins tend to collapse (reduction in particle height) into nanoscale rings due to the presence of the inner ferrihydrite core together with partial solvation of the hydrophobic components of ferritin protein through urea interactions. The solvation of hydrophobic components of globular proteins through urea interactions is well-known from MD simulations^21−24,26,51^ and also for several other proteins, such as ubiquitin^27^ and chymotrypsin.^22^ The removal of urea (4, 6, and 8 M) through repeated flushing on the graphene surface with pure water and reimaging the ferritins did not show any evidence for reversibility of the partially denatured states, indicating that the morphological changes of ferritin proteins observed on the surface of graphene induced by urea at 4, 6, and 8 M concentration were irreversible.

Although the AFM measurements provide information at a single-protein level on urea-driven unfolded states of ferritin, they still lack the time resolution to capture the effect of urea on ferritin at a solvated interface. To address this issue and quantify interactions between urea and ferritins at experimentally inaccessible nanosecond time scales, we rely on MD computer simulations of ferritin in 8 M urea. Figure 3d is a plot of the population of internal H-bonds in a ferritin subunit computed over 66 ns of free molecular dynamics showing the decreased stability of ferritin in concentrated urea when compared to control simulations of ferritin in pure water (see Figures S7–S12 for more details of the MD simulations). The computed reduction of stability for ferritin in 8 M urea vs pure water was 23 ± 3 kcal/mol, composed of penalties in ferritin conformation and ferritin residue–residue contacts. MD simulations yield a ferritin conformational penalty of 7 ± 2 kcal/mol and the ferritin contacts penalty arising from vdW and electrostatic residue–residue contacts to be 16 ± 3 kcal/mol, composed of 11 ± 3 kcal/mol from electrostatics (mainly loss in H-bonds) and a minor contribution of 5 ±1 kcal/mol from decreased vdW contacts.

The simulations further reveal that urea destabilizes ferritin both by directly coordinating with ferritin residues and by disrupting the H-bonding network in the protein solvation shell, as shown in the representative snapshot from MD simulations in the Figure 3d inset. The increased protein disorder in 8 M urea was evaluated by quantifying the root-mean-square fluctuations (RMSF) of a ferritin subunit during 66 ns of molecular simulation as shown in Figure 3e. Note that we use here a fully classical model to describe the van der Waals interactions at the solvated protein–graphene interface. While dispersion-corrected quantum models for molecule adsorption on graphene tend to agree well with classical models in terms of the van der Waals binding energies,^52^ future quantum models could incorporate the effect of substrate SiC and test for any screening of the van der Waals energy.^53^

### STEM Imaging of Urea Effects on Ferritin Proteins Encapsulated in Graphene Liquid Cells (GLCs)

**Figure 4:**
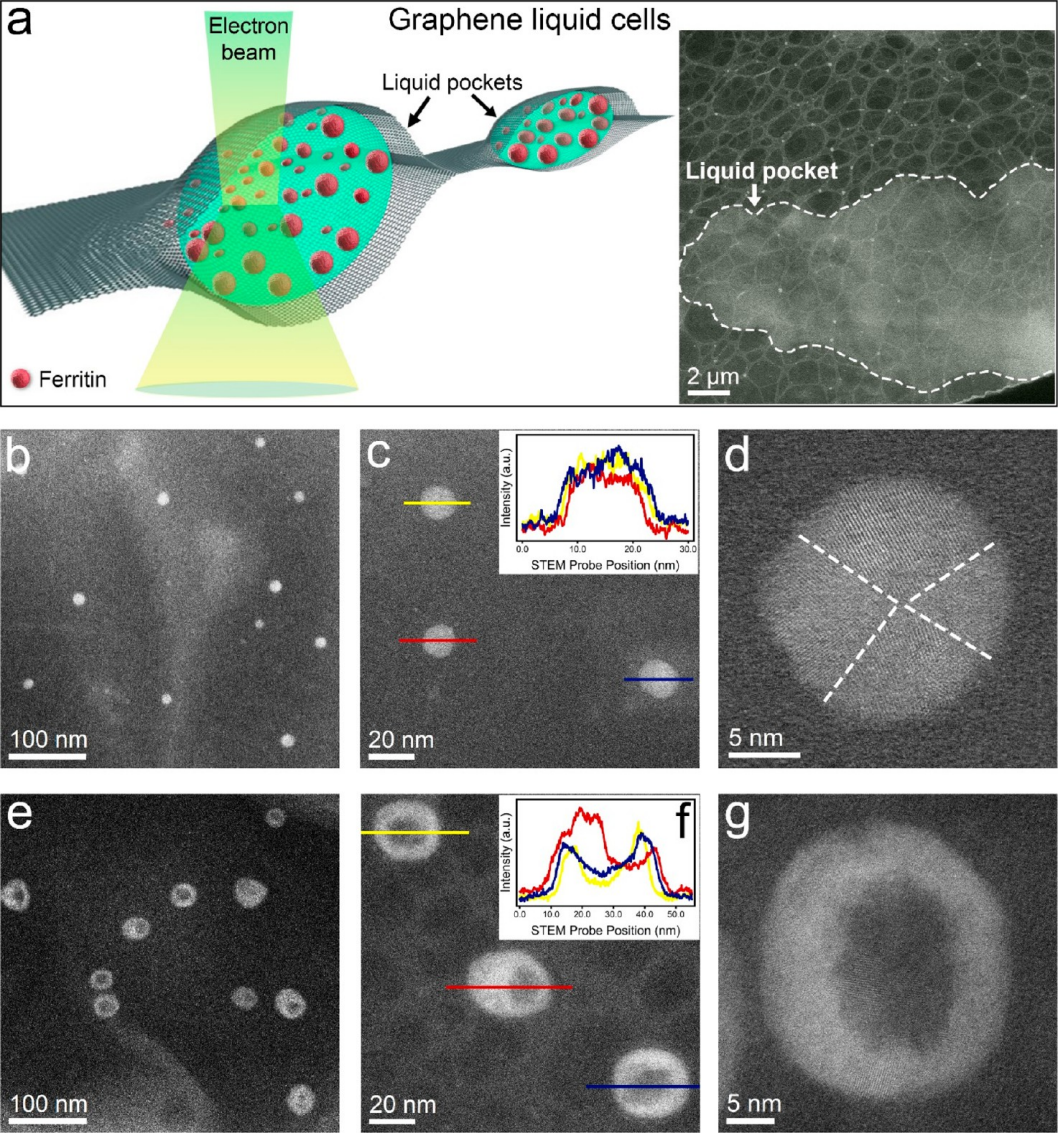
Scanning transmission electron microscopy (STEM) imaging of folded ferritin proteins and ferritin nanorings using graphene liquid cells (GLC). (a) Schematic illustration of GLCs, in which liquid containing holo-ferritin proteins is encapsulated between two graphene membranes for STEM observations. Right: the low-magnification image of a typical micrometer-sized GLC. The overlaid dashed line highlights the high-contrast liquid pocket. (b, c) Large-area images of isolated ferritin proteins in a GLC. The top right inset in panel c shows the individual line profiles extracted along the ferritin proteins marked by the image’s overlaid yellow, red, and blue lines. (d) A high-magnification image of a ferritin protein shows atomic lattice resolution and the 4-fold symmetry arrangement of the subunits marked by the overlaid dotted lines. (e, f) Large-area images of isolated ferritin nanorings in a GLC approximately 10 min after 6 M urea exposure. The top right inset in panel f shows the individual line profiles extracted along the ferritin proteins marked by the overlaid yellow, red, and blue lines in the image. (g) High-magnification image of a ferritin nanoring showing atomic lattice resolution.

To address the question of whether the formation of ferritin nanorings is influenced by the DLC-coated Si AFM probe, we performed STEM measurements (see Supporting Information Section S4 for details on the STEM setup and imaging protocols) on ferritin proteins confined in micrometer-sized GLCs (see Figure 4a). This GLC-STEM approach was demonstrated to be suitable for *in situ* observation of various phenomena, such as biomineralization of ferritins^54^ and nanocrystal growth.^55^ A low-magnification STEM image of a GLC pocket containing ferritin proteins is shown on the right side of Figure 4a (see Figure S12 for details on the GLC sample preparation). Figure 4b is a large-area STEM image of ferritin proteins enclosed in a water pocket. The spherical shape of the ferritins is best visualized in the magnified image in Figure 4c. Here, the diameters of three individual ferritin proteins are obtained from the line profiles (shown in the inset) extracted along the yellow, red, and blue lines. In addition, using GLC-STEM, we were able to acquire atomic resolution images such as the one in Figure 4d, which revealed that the ferritin proteins are composed of subunits. To investigate the effect of urea on the ferritins, we applied 20 μL of ferritin (concentration: 10 μg/mL, Type IV, CAS Number: 9007-73-2, solution in 10 mM Tris, 150 mM NaCl, pH 8.0, and 0.1% sodium azide) onto a graphene-coated TEM grid and immediately applied an additional 10 μL of urea solution (6 M). The resultant liquid was then sealed with a second graphene grid, and the GLC was immediately loaded into the STEM instrument for further imaging. Figure 4e is a large-area STEM image acquired about 10 min after the ferritin proteins were treated with urea that reveals the presence of nanoring structures. Magnified images of the ferritin proteins clearly show that the inner cavity spacing is different for each nanoring (Figure 4f and the inset with the line profiles extracted along the three nanorings). The high-resolution image in Figure 4g further demonstrates that the unfolded ferritins are polycrystalline and have a hollow center. Additional large-area and high-resolution STEM images of ferritins before and after treatment with 6 M urea are provided in Figure S13. The STEM results independently validate the previous liquid-AFM results, in which differences in the cavity of the nanorings were observed (Figure 2b–d and inset of Figure 3a) and statistically quantified (Figure 2f). These results rule out that nanorings are an artifact of AFM imaging; rather, their formation is independent of the imaging technique used.

### Contrasting GdmCl Effects on Ferritin Proteins Adsorbed on Graphene

**Figure 5:**
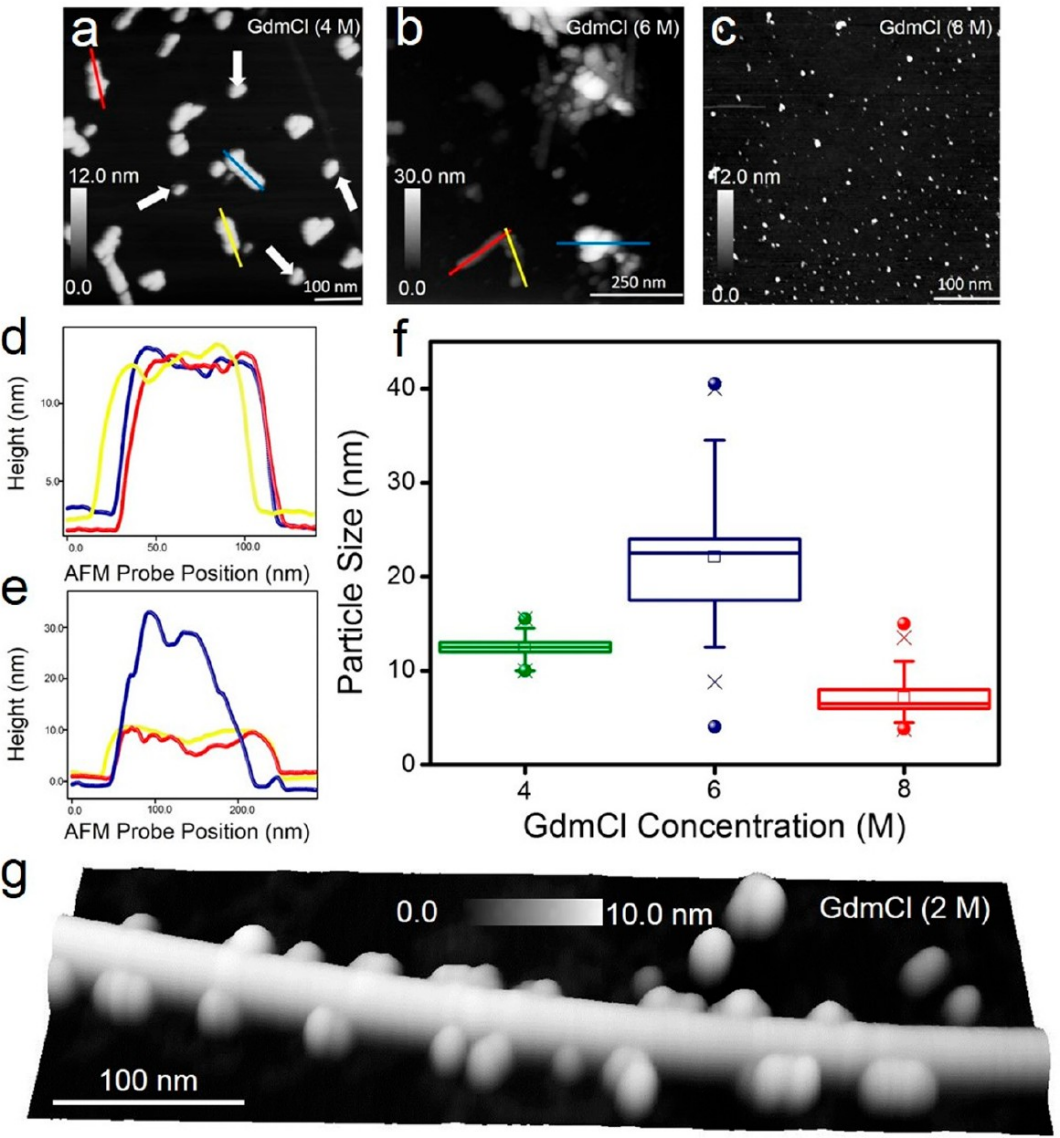
GdmCl-driven aggregation and denaturation of ferritin proteins. (a–c) Large-area AFM images showing the presence of multiprotein aggregates of ferritin in GdmCl concentrations of 4 M (panel a) and 6 M (panel b). In 8 M GdmCl ferritin proteins do not aggregate but instead form denatured single ferritin and various complexes of subunits. No evidence for denatured ferritin rings was detected when native ferritin proteins were treated with either 4, 6, or 8 M GdmCl. (d, e) Individual height profiles were measured along the red, blue, and yellow lines over aggregated ferritins indicated in panel a (4 M GdmCl) and panel b (6 M GdmCl). (f) Box chart of particle size statistics of ferritin aggregates and denatured subunits as a function of GdmCl concentration. (g) Three-dimensionally represented AFM image of ferritin aggregates which appear to crowd around an out-of-plane wrinkle on the graphene surface upon exposure to 2 M GdmCl.

**Figure 6:**
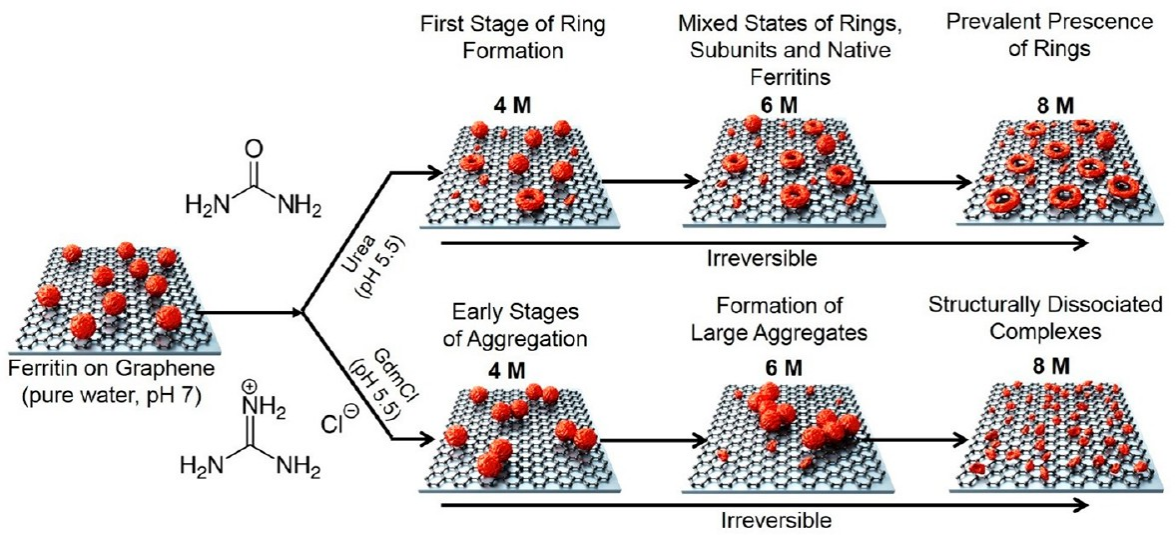
Schematic summarizing the conformational and structural changes of ferritin induced by urea and GdmCl based on the AFM imaging (objects are shown not to scale). The formation of ferritin nanorings was observed at ≥4 M concentrations of urea with an increasing abundance of rings in the distribution of natively folded ferritins, rings, and dissociated subunits. At a concentration of 8 M urea, the ferritin rings were resolved as well-spaced doughnut-shaped ring structures from the AFM images. Whereas at 4 and 6 M GdmCl ferritin protein aggregates on the surface of graphene. At a higher GdmCl concentration of 8 M, ferritin proteins were observed to dissociate completely into smaller particles. The morphological changes induced by both chemical denaturants used in this study on ferritin proteins adsorbed on graphene were observed to be irreversible.

Upon observation and quantification of the effects of interactions of urea with ferritins through combined AFM, STEM, and simulation studies, the question remains as to whether the ferritin nanorings form only upon exposure to urea or if other chemical denaturants can trigger a similar response. To address this issue, we performed control experiments on preabsorbed ferritin proteins on graphene exposed to identical concentrations of 4, 6, and 8 M GdmCl (see Supporting Information Section 2 for details on the protocols followed for GdmCl experiments). As a stronger denaturant than urea, GdmCl has been used to unfold a wide range of biomolecules, from iron-free apoferritin proteins^5^ to DNA origami structures.^37^Figure 5a–c shows large-area AFM images of ferritin in 4, 6, and 8 M GdmCl solutions, respectively. We observed no ferritin rings at any stage of these AFM measurements. Additional studies exposing ferritins to lower concentrations of GdmCl (1, 2, and 3 M) also showed no incidence of doughnut-shaped rings, in stark contrast to ferritins in urea (see Figure S6 for large-area AFM image of 2 M GdmCl effect on ferritin proteins). Exposure of ferritin proteins to 4 and 6 M GdmCl resulted in the aggregation of ferritins starting from the formation of smaller aggregates at 4 M (sectional profile analysis Figure 5d) and followed by large clusters at 6 M concentration (sectional profile analysis Figure 5e). The tendency of ferritin subunits to dissociate and reaggregate upon exposure to GdmCl was previously shown using gel electrophoresis and circular dichroism,^5^ and fluorescence spectroscopy measurements of proteins in GdmCl have also shown the tendency of GdmCl to drive protein aggregation.^16^ AFM data revealed the dissociation of ferritin proteins to subunits (Figure 5c) when ferritin proteins were exposed to 8 M GdmCl. Figure 5f is the statistical analysis in a box chart format of the size range of all of the particles (measured from the height profiles) in 4, 6, and 8 M GdmCl. Similar to urea-driven denaturation, the removal of GdmCl after ferritin denaturation by water flushing did not reverse the aggregated ferritin states back to native conformation. This result demonstrates that the GdmCl-driven unfolding of ferritins on graphene at 4, 6, and 8 M concentration is irreversible. Also, regarding the formation of aggregates of ferritins in 4 M GdmCl, we observed similar aggregated forms of ferritins even at lower concentrations, such as 2 M GdmCl. Figure 5g is a 3-D rendered AFM image of ferritin aggregates that form near an out-of-plane wrinkle on the epitaxially grown graphene film. Finally, the differences measured by AFM in this study in the unfolded states of ferritins upon exposure to 4, 6, and 8 M urea and GdmCl are summarized schematically in Figure 6.

### Urea Effects on Apoferritin Proteins Resolved Using Liquid AFM

**Figure 7:**
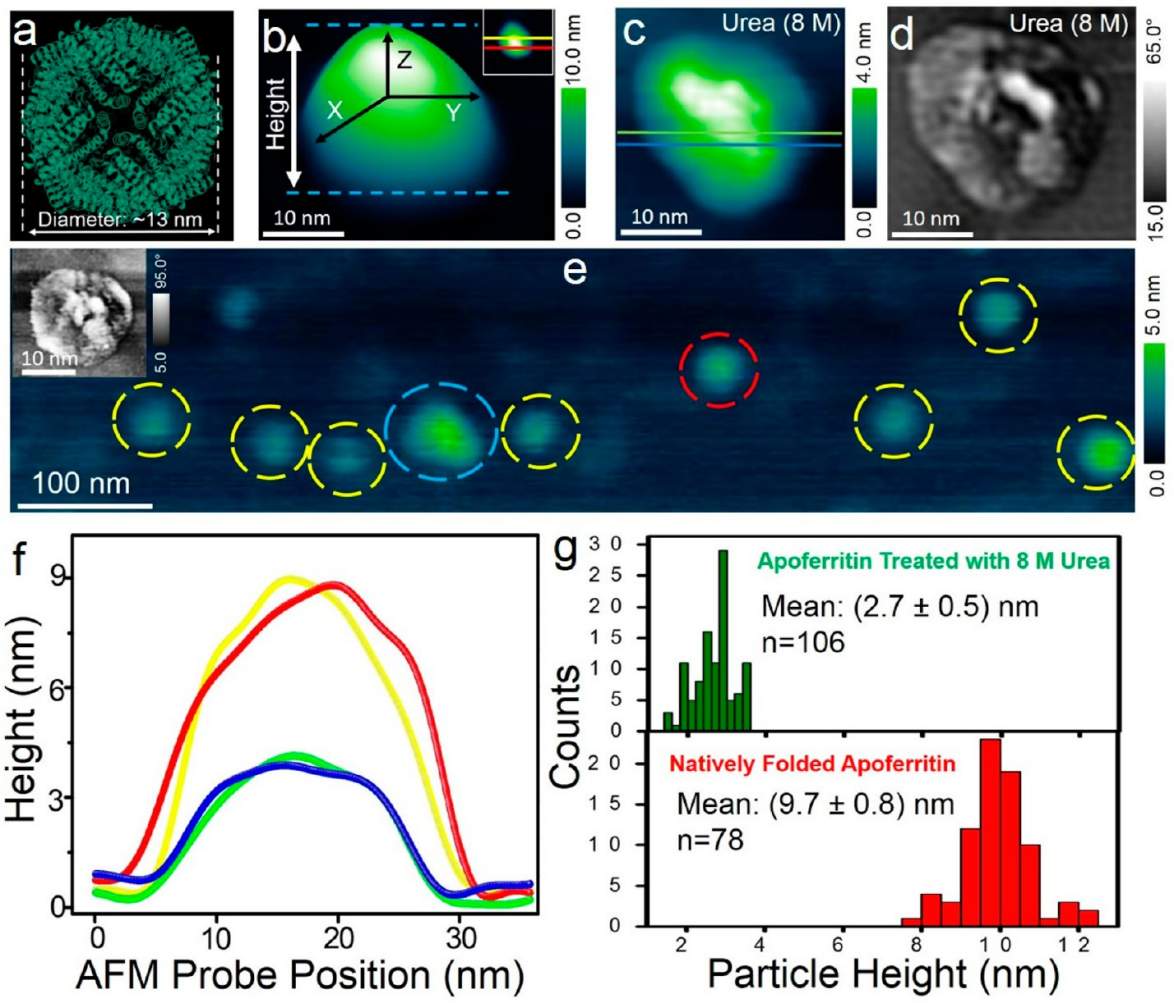
Quantifying the effect of 8 M urea on apoferritin proteins. (a) X-ray diffraction structure of horse spleen apoferritin at 1.5 Å resolution (PDB identifier: 2W0O(56)). (b) Three-dimensionally represented tapping mode AFM image of single-apoferritin protein resolved on graphene buffer–aqueous solution interface. Inset in panel a is a two-dimensional image of the same protein shown in 3-D format for cross-sectional profile analysis. (c, d) Simultaneously acquired height and phase-contrast AFM image showing an apoferritin protein of reduced height in an 8 M urea environment. The natively folded apoferritin proteins on the graphene sample were placed within the liquid-cell sample holder in the AFM setup when 50 μL of 8 M urea solution in buffered aqueous solution at 5.5 pH was injected through the inlet port. (e) Large-area AFM image (raw data without low-pass filtering) of apoferritin proteins in 8 M urea medium. Apoferritin particles with reduced height are highlighted with yellow circles. The particle indicated with the blue circle appears to be of higher height value than most of the surrounding particles. The inset in panel e is a phase-contrast image recorded over the apoferritin particle indicated by a red circle. (f) Height sectional profiles extracted along with individual natively folded (yellow and red traces) and along 8 M urea treated (blue and green traces) apoferritin proteins from the AFM images shown in the inset of panels b and c reveal the height differences of apoferritin as a function of their chemical environment. (g) Statistical analysis of natively folded apoferritin height of 9.7 ± 0.8 nm (bottom red histogram) and urea-treated apoferritin particle height analysis of 2.7 ± 0.5 nm (top green histogram).

After examining the role of urea and GdmCl on ferritin and confirming that only urea treatment of ferritin leads to the formation of nanoscale rings, we studied apoferritin. We exposed it to urea with the identical concentration values used in the holo-ferritin unfolding experiments. Control tests were conducted on natively folded horse spleen apoferritin proteins (structure resolved at 1.5 Å is shown in Figure 7a, PDB code: 2W0O(56)) with a known outer shell diameter of ∼13 nm^57^ (see Supporting Information Section S5 for details on apoferritin sample preparation). The natively folded apoferritin protein size and shape profile are shown from the 3-D rendered AFM image shown in Figure 7b. Treating the preadsorbed apoferritin proteins on graphene with 8 M urea resulted in particles with reduced height compared to the natively folded apoferritin proteins, as shown in simultaneously acquired height (Figure 7c) and phase-contrast (Figure 7d) AFM images. A large-area AFM image (Figure 7e) shows the reduced height of apoferritin particles compared with natively folded apoferritin particles. The sectional profiles extracted along the individual natively folded (yellow and red traces) and urea-treated apoferritins (blue and green traces) are shown in the section analysis plot (Figure 7f). A comparative quantitative distribution of particle heights before (red histogram, natively folded apoferritin particle height) and after urea treatment (green histogram) is shown in Figure 7g. These control experiments on natively folded single-apoferritins conducted in a controlled urea environment did not result in any nanoscale rings and further confirm that the formation of nanometer-sized rings is the signature of urea–ferritin interactions. Future combined simulation–experimental studies could further explore the contrast between the denaturing process of ferritin and apoferritin proteins in a urea environment to further explore the denaturing mechanism.

## Conclusion

In this first report of protein nanoring formation, we used atomic force and electron microscopy supported by MD simulations to understand how urea destabilizes ferritin. We demonstrate an *in situ* approach to monitor and quantify the effect of urea and GdmCl on single ferritin proteins retained in a hydrated state using liquid-based AFM and GLCs-based STEM. Using *in situ* microscopy operated at a solid–liquid interface, we bring to light previously unseen denatured states such as the presence of nanometer-sized rings, which were generated on graphene surfaces when natively folded ferritin proteins were exposed in a controlled manner to urea in the solution phase. The geometry of the toroidally shaped nanorings depends on the concentration of urea used, and the changes in ring height and inner cavity spacing is irreversible. The spontaneous formation of nanoscale rings could be exploited as templates if the rings can be metalized for plasmonic nanostructures.^58^ To probe more deeply into the atomic-scale mechanism of ferritin restructuring in urea, future work will focus on coarse-grained molecular dynamics simulations and atomic-scale molecular dynamics with accelerated sampling methods to advance the current models to simulate the dynamic restructuring of the full ferritin protein in urea, which may be achievable by using intensive high-performance computing.

Here we focused the MD models on the experimentally observed ferritin restructuring solution of 8 M urea in water. Alternative chaotrope concentrations and types had lower or no effect on nanoring formation. The 8 M urea models allowed us to capture the atomic-scale destabilization at the submicrosecond time scale of the simulations. In general, the chaotrope-induced denaturation follows a sigmoidal response to chaotrope concentration with linear destabilization energy region that shifts according to chaotrope type and physical conditions.^60,61^ Future models with alternative urea concentrations can be built simply by scaling the ratio of urea to water in the solvent phase (Figure S12), and GdmCl/water models^59^ can be used for future comparative simulation studies of the different chaotropes.

Although the spatial resolution of the liquid-based AFM is limited to relatively large changes in single protein shape or dissociation into subunits, the benefits of this approach could start to address an important unmet need to investigate conformational changes and hydrophobic hydration effects that can occur during the denaturation. For example, it has been recently shown that urea can modulate growth^62^ and aggregation kinetics^63^ of amyloid beta peptides implicated in the pathology of Alzheimer’s disease,^64,65^ which we anticipate can now be visualized, and morphological changes quantified one protein at a time.

## Data Availability

All data needed to evaluate the conclusions in the paper are present in the paper and/or the Supporting Information. Additional data related to this paper may be requested from the authors.

## Supporting Information Available

Details of the liquid-AFM setup, AFM probe cleaning procedure, preparation of urea, GdmCl, ferritin, and apoferritin solutions, STEM, GLC setup, and molecular dynamics simulation methods (PDF)
