# Supplementary material for: In Situ Observation of Chemically Induced Protein Denaturation at Solvated Interfaces
Source: ACS Appl Mater Interfaces. 2023 Oct 5;15(41):48015–26. doi: 10.1021/acsami.3c10510 (PMC10591235; doi:10.1021/acsami.3c10510)
Supplement: Supplementary file 1 — am3c10510_si_001.pdf [file am3c10510_si_001.pdf]

Supporting Information

In situ Observation of Chemically-induced Protein Denaturation at Solvated Interfaces

Peter Niraj Nirmalraj*[1,4], Marta D. Rossell[2], Walid Dachraoui[2], Damien Thompson[3] and Michael Mayer*[4].

1. Transport at Nanoscale Interfaces Laboratory, Swiss Federal Laboratories for Materials Science and Technology, Überlandstrasse 129, 8600, Dübendorf, Switzerland. 2. Electron Microscopy Center, Swiss Federal Laboratories for Materials Science and Technology, Überlandstrasse 129, 8600, Dübendorf, Switzerland. 3. Department of Physics, Bernal Institute, University of Limerick, V94T9PX, Ireland. 4. Adolphe Merkle Institute, University of Fribourg, Chemin des Verdiers 4, CH-1700 Fribourg, Switzerland.

*Corresponding authors. Corresponding authors email: peter.nirmalra@empa.ch and michal.mayer@unifr.ch

Contents

Section S1: AFM imaging of proteins adsorbed on graphene in pure water

Figure S1: Liquid-based atomic force microscopy experimental setup

Section S2: Preparation of solutions with chaotropic agents

Figure S2: Contamination of DLC-coated AFM probe and verification of the cleaning procedure

Figure S3: Stability of nanorings on gold surface

Figure S4: Ferritin subunit analysis

Figure S5: Plot of nanoring cavity spacing as a function of urea concentration

Figure S6: GdmCl (2 M) induced aggregation of ferritins

Section S3: Molecular dynamics simulations

Figure S7. Ferritin peptide unit sampled in water and in 8 M urea mixed water-urea solvent

Figure S8. Computed heat maps of RMSF (Å) for ferritin peptide in water and 8 M urea

Fig. S9: Interaction of full ferritin protein with an alkylamine SAM-coated graphene surface

Table S1: Computed weak binding interaction energies of ferritin on SAM-coated graphene and graphene

Fig. S10: Full model view of the ferritin-graphene complex that forms the strong interface

Fig. S11: The 8M urea solvent simulation cell used to model ferritin in the chaotropic environment.

Section S4: Details on liquid-TEM-based analysis of ferritin nanoring formation

Figure S12: Encapsulated liquid

Figure S13: Graphene liquid cell STEM images of ferritin proteins in water and in 6 M urea

Section S5: Preparation of apoferritin sample solution

Section S1: AFM imaging of proteins adsorbed on graphene in pure water: Liquid-based atomic force microscopy was performed using a JPK Nanowizard II and Bruker Multimode 8 AFM equipped with a flow cell. For the AFM probe, a diamond-like carbon (DLC) tip with Aluminium reflex coating (Tap150DLC-10**,** for soft tapping mode) resonant frequency: 150 kHz, force constant: 5 N/m, tip apex radius: ~10 nm was used in tapping mode. Immediately after receiving the AFM tips in a gel pack from Budget Sensors, the tips were transferred to a standard plastic sample holder to avoid tip contamination by the gel pack. Before inserting the AFM probe in the holder, the AFM tip was cleaned by rinsing in acetone for 30 s followed by rinsing in isopropanol for ~1 min followed by blow-drying with compressed nitrogen or air.

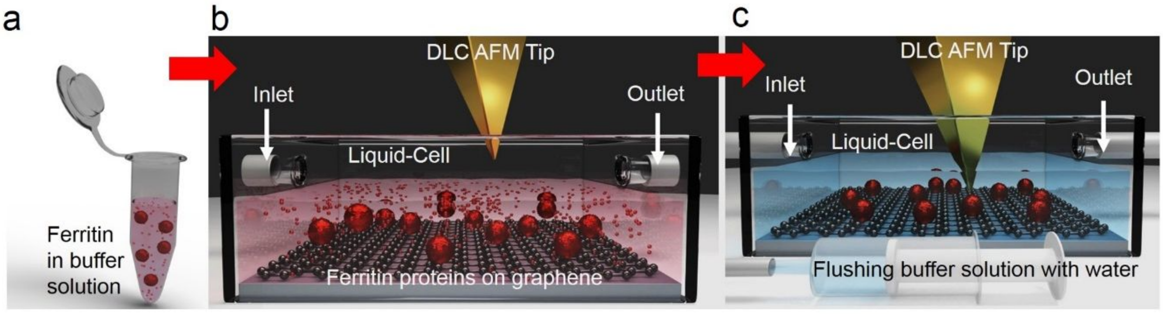

Figure S1: Liquid-based atomic force microscopy experimental setup. (a) Ferritin in buffer salt solution. **(b)** Schematic of ferritins deposited from buffered aqueous solution on graphene placed within a perfusion-type liquid cell (objects are shown not to scale). **(c)** The liquid cell is then gently flushed with pure water to remove the buffer solution and buffer salts from the graphene surface after some time of ~2 min after injection of ferritins. An ultrasharp AFM probe is immersed into the water medium, tuned, and then engaged in tapping mode to image the ferritins adsorbed at the graphene water interface.

After mounting the AFM probe in the tip holder, 20 µL volume of ferritin (concentration: 10 µg/mL, Type IV, CAS Number: 9007-73-2, solution in 10 mM Tris, 150 mM NaCl, pH 8.0, and 0.1% sodium azide) was injected through the inlet port in the liquid-cell holder containing epitaxially grown graphene at room temperature. Before deposition of ferritin solution, the epitaxially grown graphene (purchased from Graphene Supermarket) samples were thoroughly cleaned (acetone rinsing followed by isopropyl alcohol rinsing and blow drying with N_2) and the surface quality was checked using an AFM. After ~2 min the liquid cell was gently flushed with pure water and the AFM immersed and tuned in the pure water medium to generate the AFM images shown in Fig. 1C and 1D of the main manuscript. The resonant frequency of the probe, when measured in the aqueous buffer solution, was ~100 kHz, which is a damping of the original resonant frequency (150 kHz) of the cantilever due to the water medium. After the tuning procedure, the probe was brought near the sample and the tip operated in tapping mode. The values for the standard deviation reported for the diameter of native ferritins were calculated from measurements performed with ten AFM tips from the same batch purchased from Budget Sensors.

Section S2: Preparation of solutions with chaotropic agents: Urea was purchased in powder form from Sigma Aldrich (CAS number: 57-13-6), dissolved in pure water, and tris buffer (100 mM, pH 7.4 purchased from Sigma Aldrich, CAS number: 77-86-1) and the pH of the urea (8 M) stock solution was adjusted to 5.5 pH. A stir plate was used to solubilize the 8 M urea solution overnight followed by filtration before usage. The stock solutions were all used within two weeks of preparation. For the preparation of the GdmCl solution, GdmCl was purchased in powder form from Sigma Aldrich (CAS number: 50-01-1) and solubilized in pure water and tris buffer ((100 mM, pH 7.4), and the pH of the GdmCl stock solution was adjusted to 5.5 through the addition of NaOH. To solubilize the 8 M GdmCl solution it was heated up to 35°C for ~30 minutes as recommended by the commercial supplier. All the pH measurements were made using a calibrated Mettler Toledo pH meter.

<u>AFM Imaging of proteins in solutions with Urea and GdmCl:</u> For AFM measurements in urea (4, 6, and 8 M) and GdmCl (4, 6, and 8 M), the protocol followed was to inject 10 μL of the chemical denaturant (urea solution, pH 5.5 and GdmCl, pH 5.5) in separate experiments within the liquid-cell where previously ferritin (20 μL volume) was injected onto the graphene surface. The AFM probe was tuned in the chemical denaturant medium and then engaged in tapping mode to resolve the effect of the chemical denaturants on the adsorbed ferritin. Usually, it takes ~30 s for this procedure (tip tuning and engaging in tapping mode) to complete followed by establishing stable and reliable AFM imaging of the denatured ferritin states. Until now, we have not been able to examine the ferritin rings (in the case of urea studies) formed in less ~ 1 min of urea exposure. However, the collapsed rings may form well before that period, however instrumental operation time and limited scanning speed of the AFM impede the ability to resolve in real-time the transition of globular-shaped ferritins to collapsed rings. Capturing the time-elapsed imaging of protein folding and resolving in real-time the effect of chemical denaturants on proteins should in principle be possible through a video-rate AFM operating at a scanning speed of 10 frames per second[1]. All the AFM images reported in this work were analyzed using Gwyddion software (version 2.51).

<u>Cleaning of DLC-coated AFM probes after exposure to urea and GdmCl:</u> Immersing and operating the DLC AFM probe in urea and GdmCl at all concentrations (2-8 M) for longer than 40-60 min resulted in double tip effects. These contamination-induced effects were unavoidable. On the other hand, a bare Si tip or metal-coated AFM tips (Au, Pt, Pt-Ir) did not provide reliable imaging beyond 5-10 min in urea and GdmCl solutions due to tip contamination from the encompassing medium. Removing the tip from the holder, rigorously cleaning and reusing it for imaging, showed that the cleaning procedure for the bare Si and metal-coated tips were not effective. The same procedure when used for the DLC-coated AFM tips showed that the procedure worked and that it can be used routinely to clean these expensive tips for reuse in urea and GdmCl.

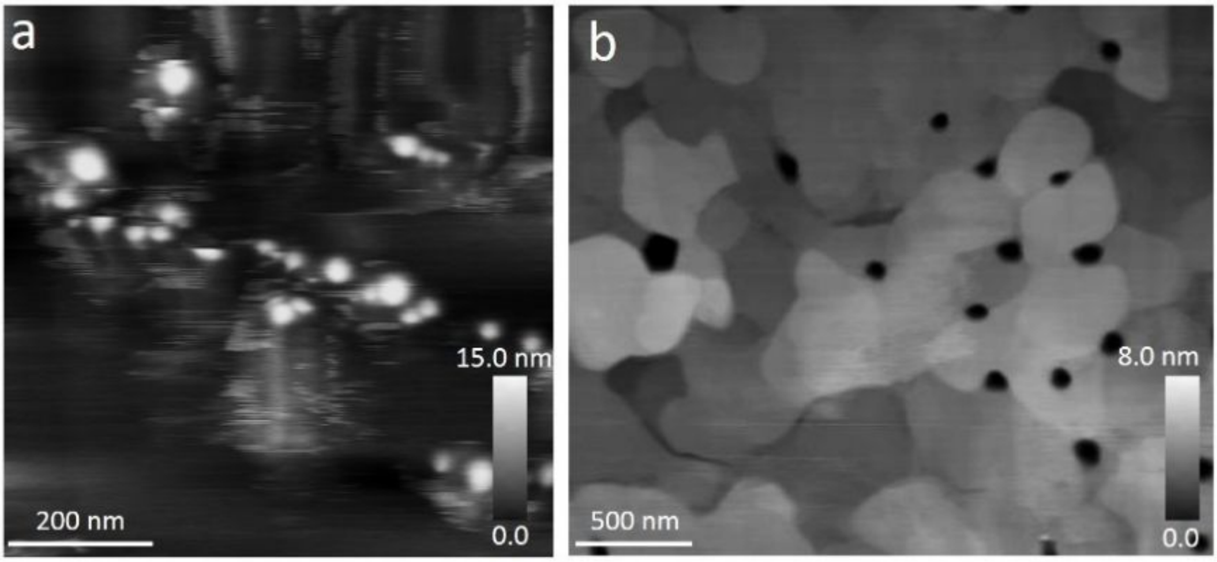

Figure S2: Contamination of DLC-coated AFM probe and verification of the cleaning procedure. (a) A typical example of a contaminated DLC AFM tip when used to probe the adsorbed ferritins in urea (8 M). The image was taken ~45 min of exposure in urea. (b) AFM image of Au(111) surface resolved using a cleaned DLC AFM probe using the steps detailed above. The surface texture of the Au(111) surface confirms that the cleaning procedure was successful and such cleaned tips were reused for imaging ferritins adsorbed on graphene in urea and GdmCl environments.

The cleaning procedure is as follows:

1. In a freshly prepared piranha solution (3:1 mixture of concentrated sulphuric acid and 30% hydrogen peroxide, where hydrogen peroxide is first added to the sulphuric acid) the contaminated DLC probe, which was confirmed from the double tip and streaking effects (**Fig, S2a)** is gently immersed for 5 minutes. A longer exposure could induce etch pits on the DLC coating.
2. The DLC AFM probe in then cleaned in DI water, acetone, and isopropyl alcohol to remove possible residues from the piranha solution from the AFM probe.
3. The AFM probe is then immediately blown dry with compressed N_2 gas.
4. The quality of the as-cleaned AFM tip is then checked on a clean Au(111) surface under ambient conditions and upon confirming the quality of the tips (lack of double tip effects and resolving atomically flat gold terraces, **Fig. S2**) they are reused for imaging proteins in urea and GdmCl solutions.
5. From our experiments, we confirm that a single DLC-coated AFM tip can be cleaned and reused for protein imaging about 5-8 times using the above steps.

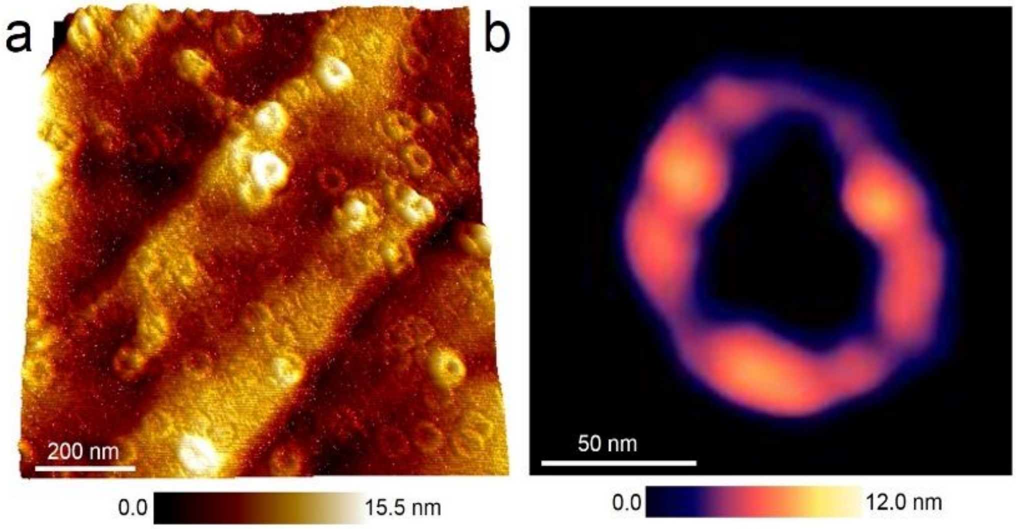

Figure S3: Stability of nanorings on gold surface. (a) Large-area 3D AFM image of a nanorings formed on Au(111) after treating ferritin proteins with 6 M urea. (b) High-resolution image of a nanoring adsorbed on Au(111). After removing urea solution from the liquid-cell and gently flushing the gold surface with clean water, the rings remaining stable for over several days both in liquid medium and upon air-drying.

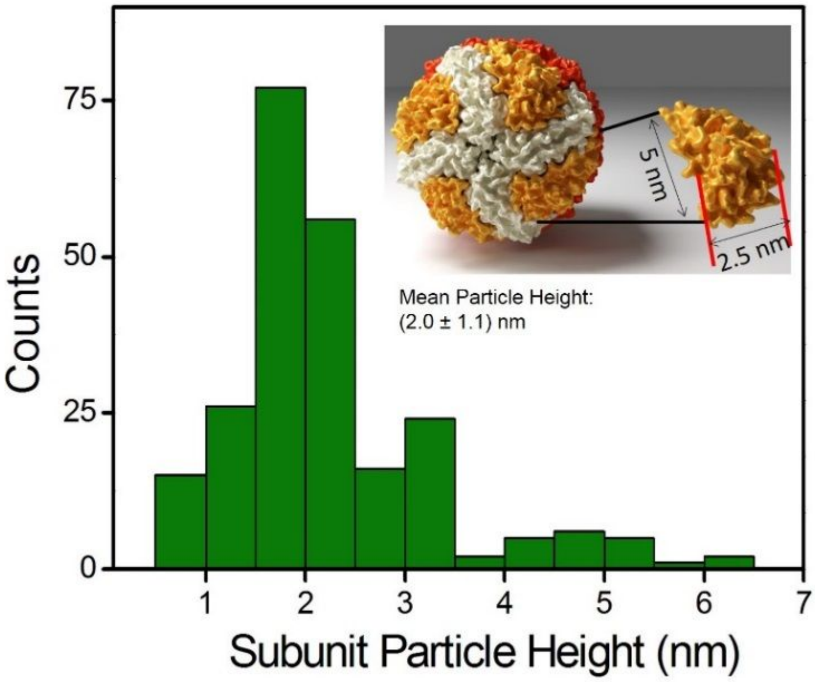

Figure S4: Ferritin subunit size analysis: The size of a single ferritin subunit has been experimentally determined to be ~5 nm in length and ~2.5 nm in width[2-3]. From the AFM data (particles indicated by green arrow in **Fig.2c** shown in the main manuscript) we measure a mixture of broken subunits, single subunits, and subunit aggregates. The denatured ferritin particles were observed when native ferritins were exposed to 4, 6, and 8 M urea concentrations.

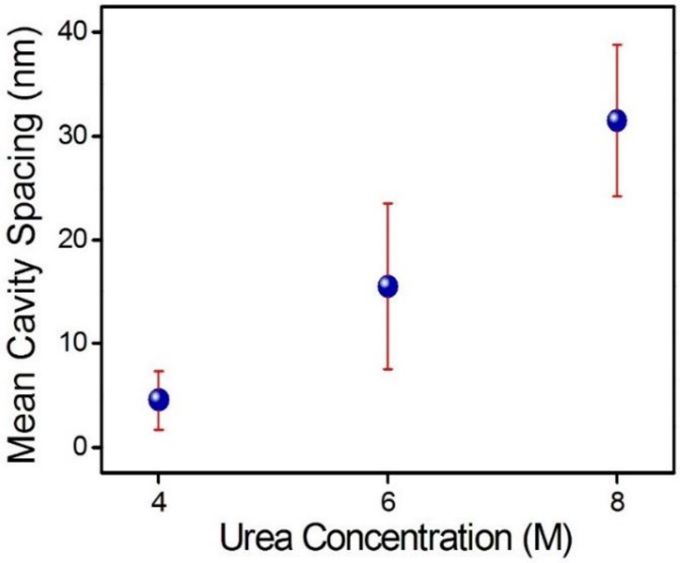

Figure S5: Plot of nanoring cavity spacing as a function of urea concentration. The measured inner cavity spacing of the ferritin nanorings is observed to scale with urea concentration. The AFM images and the sectional profile traces for the nanorings formed when native ferritins are exposed to 4, 6, and 8 M urea are provided in the main manuscript (**Fig 2b-d** and **Fig. 3a-b**). The statistical plot of ring cavity spacing for nanorings formed in 8 M urea is provided in Fig. 3b in the main manuscript.

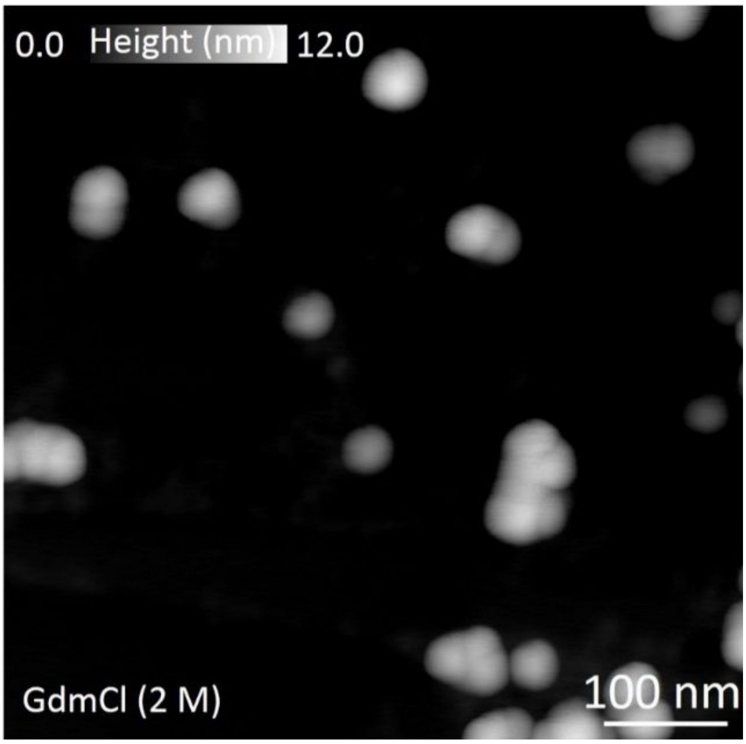

Figure S6: GdmCl (2 M) induced aggregation of ferritins. The AFM data shows that the ferritin proteins tend to aggregate even at GdmCl concentrations as low as 2 M. Single ferritin proteins are also present together with the aggregates on the surface of graphene. In contrast to urea, we did not observe any nanoscale ferritin rings at GdmCl concentrations from 2-8 M, pH 5.5. For AFM data on ferritin proteins exposed to 4-8 M GdmCl, refer to **Fig 5** in the main manuscript.

Section S3: Molecular dynamics simulations

Fully atomistic molecular dynamics simulations were performed using the NAMD code[4]. The CHARMM force field was used[5] for protein and solvent molecules (Fig. S7). All simulation cells were neutralized by adding an appropriate number of Na^+ or Cl^- ions. After 5000 steps of minimization using the conjugate gradient method, each system was equilibrated and thermalized to 300 K over six consecutive steps (1 ns each), with the force constants of the protein backbone and sidechain atoms gradually scaled from 1000 to 0 kJ/(mol nm^2) and 500 to 0 kJ/(mol rad^2), respectively.

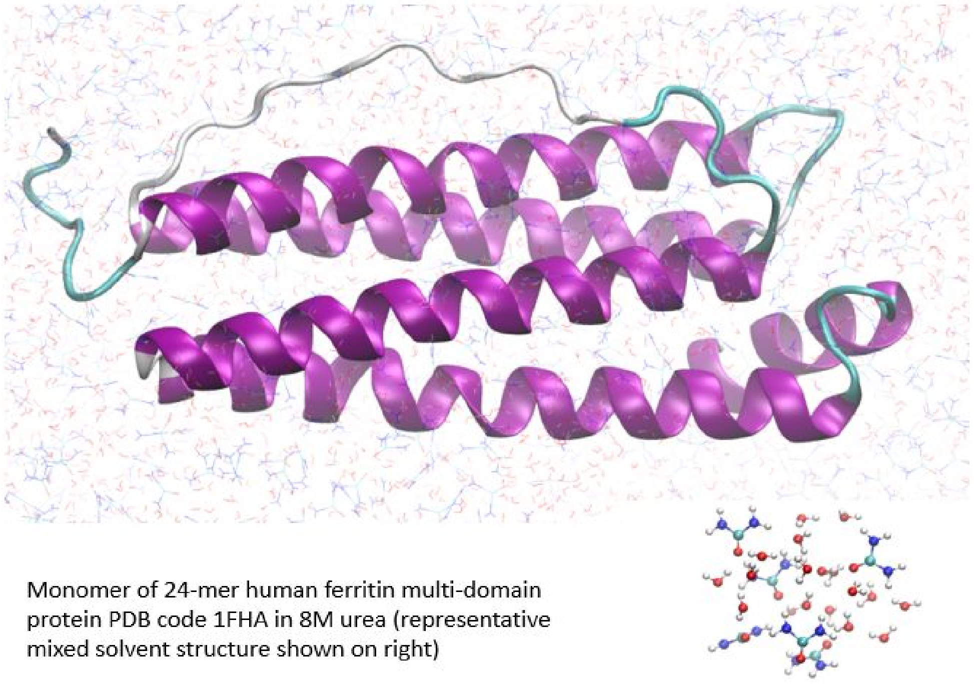

Figure S7. Ferritin peptide unit sampled in pure water and 8 M urea (mixed water-urea solvent). The peptide is shown in cartoon representation with solvent molecules shown as lines with the bottom righthand inset showing water-urea mixed clusters present in 8M urea in a ball-and-stick representation. Carbon atoms are colored cyan, nitrogens are blue, oxygens are red and hydrogens white.

Periodic boundary conditions were applied with long-range electrostatic interactions treated using the particle-mesh Ewald (PME) method. Each simulation cell was maintained at 300K and 1 atm using Langevin dynamics with an integration time step of 2 fs.

Molecular dynamics simulations were also used to compare the stability of a ferritin monomer (one unit of the 24-mer sampled at the graphene water interface, above) in pure water and 8M urea (water/urea mixed solvent). The 8 M urea mixed solvent box was constructed and modeled using literature protocols[6]. The subunit was sampled for 66 ns of equilibrated room temperature molecular dynamics in each solvent (**Figure S7** and main text **Figure 3d**). Shown in **Figure S8** are heat maps of the computed

Root Mean Square Fluctuations of the peptide Cα atoms in pure water and 8 M urea showing increased flexibility and disordering of the ferritin unit in 8 M urea (as plotted in main text **Figure 3d**).

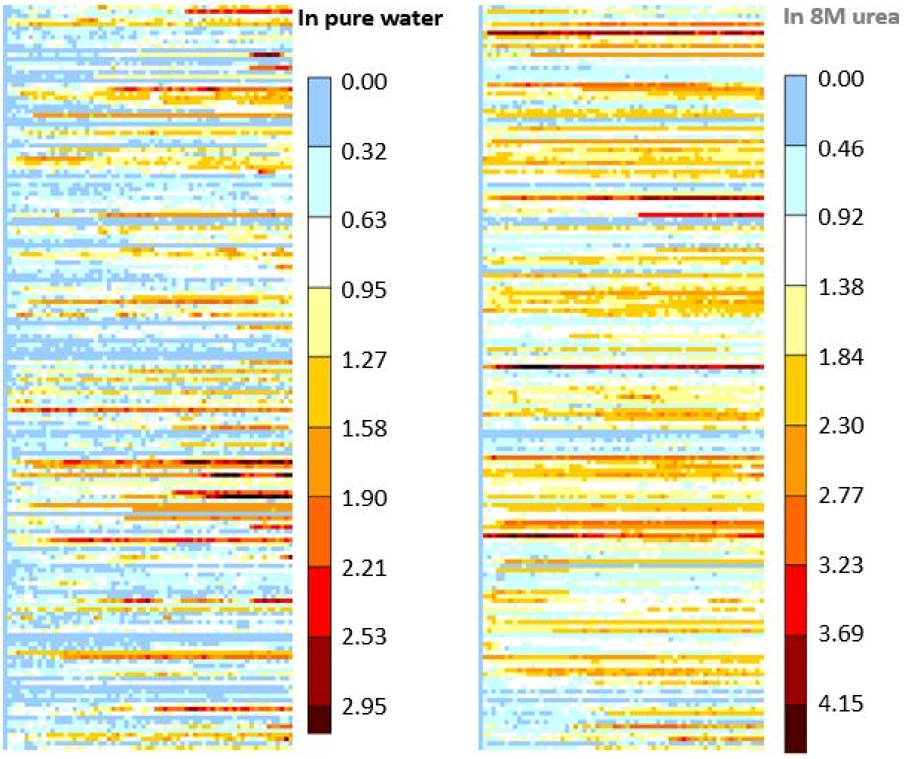

Figure S8. Computed heat maps of RMSF (Å) for ferritin peptide in water and in 8 M urea. The vertical axis shows computed values (from bottom to top) for residue numbers 1 to 183 and the horizontal axis shows sampling time from 0 to 66 ns of equilibrated room temperature dynamics.

In addition to the long molecular dynamics computer simulations of the ferritin sub-unit in water and urea (Fig. S7), simulations were performed in water for 100 ns to monitor protein-surface contacts as the full 24-mer ferritin protein interacts with single-layer graphene, *via* two alternative protein faces at the entrance to the hydrophobic four-fold channel and the entrance to the hydrophilic three-fold channel. In both starting orientations, ferritin makes only very weak, transient interactions with bare uncoated graphene when approaching the surface as a fully solvated single protein in bulk water. Ferritin makes weak, short-lived contacts by Lys124 giving small time-averaged vdW stabilization of -4 ± 1 kcal/mol. Similarly for control simulations using an alkylamine-coated graphene substrate[7], we see only transient ferritin–surface interactions (**Figure S9, Table S1**).

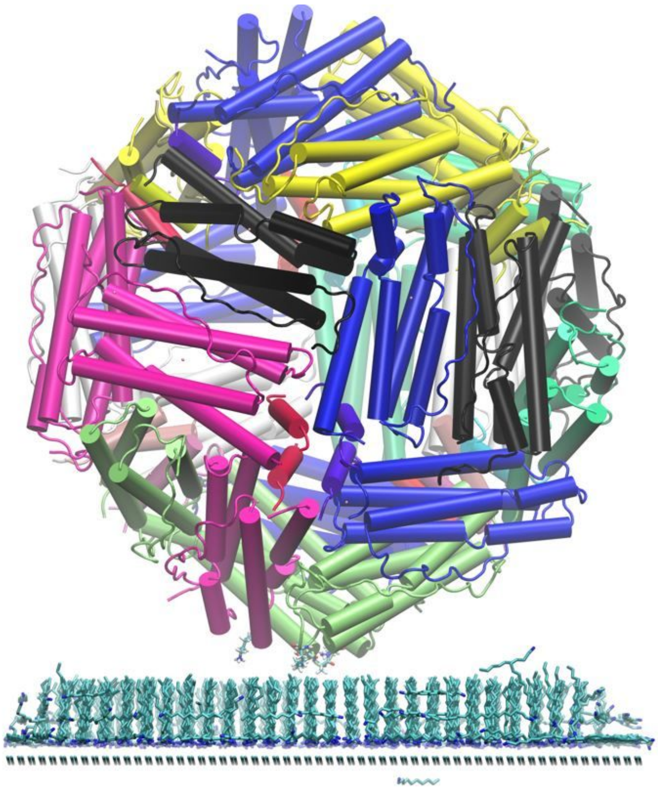

Fig. S9: Interaction of full ferritin protein with an alkylamine SAM-coated graphene surface. We modelled this alternative to bare graphene to explore the possibility of designing an alternative adsorption platform for ferritin imaging on graphene. As shown in Fig. S10 and main text Fig.1, a strong contact was obtained on bare graphene by mimicking macromolecular crowding and utilizing the effect of a large local concentration of ferritin to drive formation of a tighter, more long-lived protein adsorption mode on bare graphene.

Table S1: Computed weak binding interaction energies of ferritin on SAM-coated graphene and bare graphene. Molecular dynamics computer simulations were performed in water for 25 nanoseconds for each binding mode, to monitor protein-surface contacts as ferritin adsorbs, via two alternative protein faces at the entrance to the hydrophobic four-fold channel and at the entrance to the hydrophilic three-fold channel. The time-averaged energies are taken from the final 10 ns of dynamics from statistically independent structures obtained by sampling every 100 picoseconds.

Binding mode	4-fold on SAM-coated graphene	3-fold on SAM-coated graphene	4-fold on bare graphene	3-fold on bare graphene
Electrostatic interaction energy (kcal/mol)	-18.2 (2.1)	-10.8 (1.2)	N/A	N/A
van der Waals interaction energy (kcal/mol)	-1.7 (0.4)	-17.0 (2.4)	-0.5 (0.2)	-3.8 (1.2)
Total interaction energy (kcal/mol)	-19.9 (2.1)	-27.8 (2.6)	-0.5 (0.2)	-3.8 (1.2)

To better model the experimental configuration and capture ferritin adsorption at experimental timescales at a high local concentration of ferritins on the graphene, we performed a further set of simulations using tight, partially desolvated starting protein-surface interfaces (**Figure S10**). In these simulations, ferritin makes a stable large-area contact summed over multiple weakly-bound groups (main text **Figure 1e**) to give a time-averaged van der Waals binding energy of (-88 ± 5) kcal/mol.

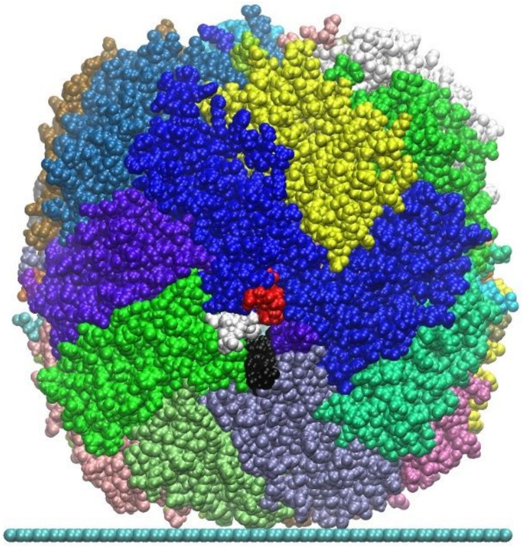

Fig. S10: Full model view of the ferritin-graphene complex that forms the strong interface. Zoom-in of the interface is shown in main text Fig.1. Here the full ferritin protein is visualised as space-filling atoms and the component protein monomers are colored by sub-unit. Solvent molecules are omitted.

On average 9 ± 1 residues of the adsorbed ferritin face makes contact with the graphene surface, creating individually-weak binding energies of approximately -10 kcal/mol, or 430 meV each, with a small full-protein conformational penalty +10 ± 4 kcal/mol stemming mainly from dihedral angle bending of the four glutamates at the entrance to the four-fold channel.

In future work, alternative solvent models can be created by changing the ratio of urea to water and re-optimising the unit cell to its equilibrium density (**Figure S11**). Similarly, the type of chaotrope molecule can be changed by replacing urea with, for example, guanidinium chloride, and re-optimising the cell.

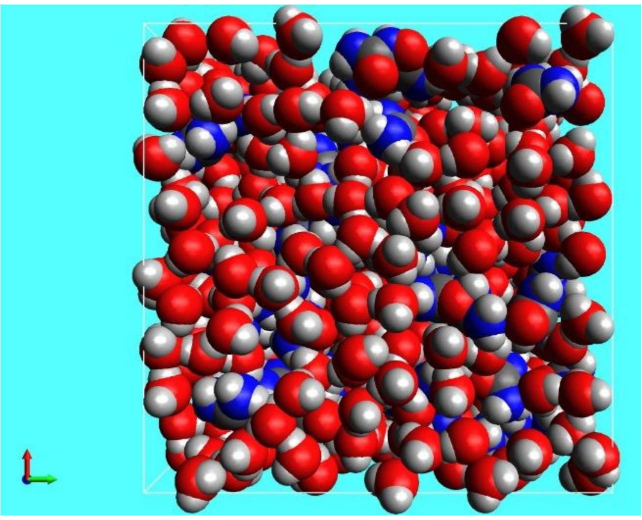

Fig. S11: The 8M urea solvent simulation cell used to model ferritin in the chaotropic environment. Water and urea molecules are shown as space-filling atoms scaled according to their van der Waals's radii.

Section S4: Liquid-TEM-based analysis of ferritin nanoring formation

STEM imaging of proteins in graphene liquid cells: Liquid-based scanning transmission electron microscopy was performed at room temperature using a probe-corrected FEI Titan Themis microscope operated at 300 kV and setting a probe convergence semi-angle of 18 rad (**Figure S12**, **Figure S13**). To limit electron beam damage, low-dose annular dark field (ADF) STEM imaging was performed at an electron probe current of <5 pA in combination with collecting semi-angles of 18–108 mrad for the annular dark-field detector. ADF-STEM images of 2048 x 2048 pixels at a pixel dwell time of 0.8 and 1.6 μs were acquired. Thus, for the low-magnification images, the electron dose rate was <1 electron/$Å^2$·s, while the dose rate for the high-magnification images was between 29 and 34 electrons/$Å^2$·s.

Graphene liquid cell (GLC) fabrication procedure: The GLC is prepared by a direct grid-on-grid sandwich technique[8]. The method is described in our previous publications[9-10].

Verification of liquid presence in the GLCs: The presence of the aqueous solution inside the pockets during imaging is confirmed by the following observations:

i- Bubbles formation: When the electron beam is focused on the liquid cells, bubbles can be seen around the ferritin nanoparticles (Fig. S12a), which is a very common phenomenon in liquid cell experiments[11-12].

ii- Contrast difference inside and outside the liquid pocket: The image contrast is highest in the middle of the liquid pockets and gradually decreases towards the edges due to the reduction of the liquid thickness. Consequently, as electron scattering is weaker at the edge of the pockets, the image quality improves and images of the ferritin structures obtained (with the same imaging conditions) from these thinner liquid areas are better resolved. This confirms the presence of solution in the liquid pockets (more details in **Figure S12b**).

Taken together, these tests clearly indicate that all the results of our experiments are obtained in liquid phase.

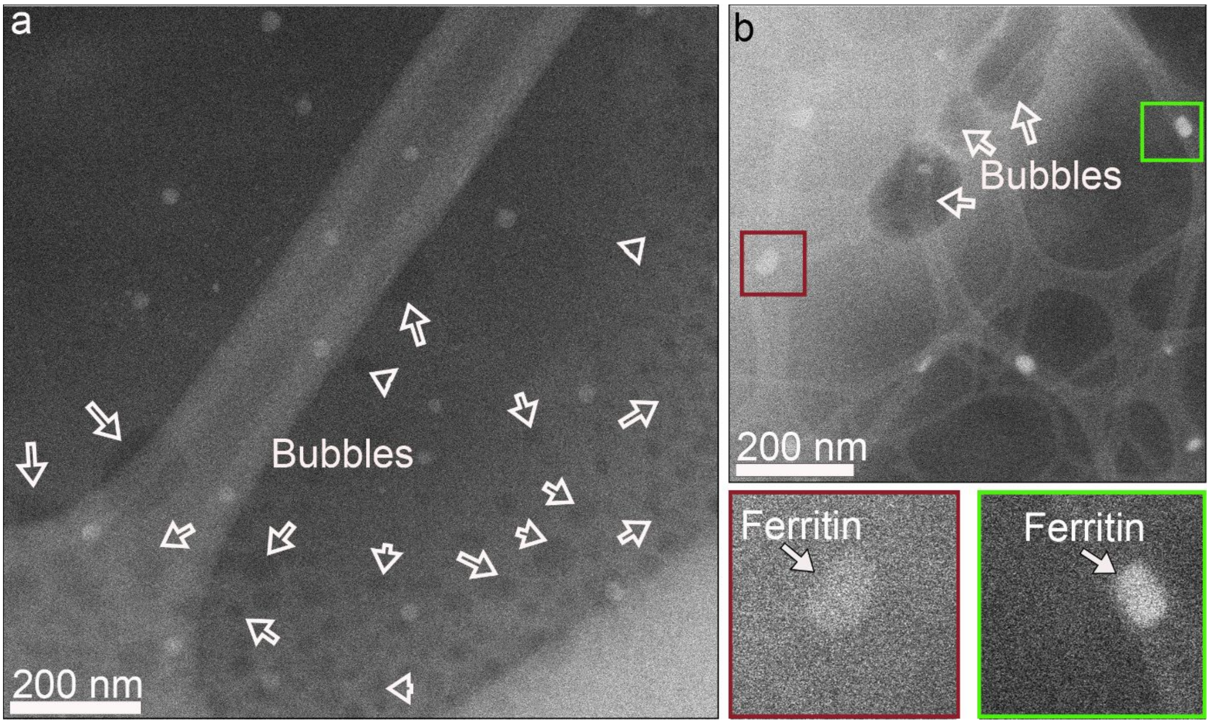

Figure S12: Encapsulated liquid. (a) Representative ADF-STEM image of the liquid pocket showing the formation of bubbles (indicated by white arrows) in the surrounding area of ferritin nanoparticles. **(b)** Representative ADF-STEM image of the liquid pocket showing the presence of bubbles. Two ferritin nanoparticles in the middle and at the edge of the liquid pocket are highlighted by red and green squares, respectively**.** Note that the contrast-to-noise ratio of the (green) ferritin located at the edge of the liquid pocket is higher, as the electron scattering is weaker in this region than in the center of the liquid pocket.

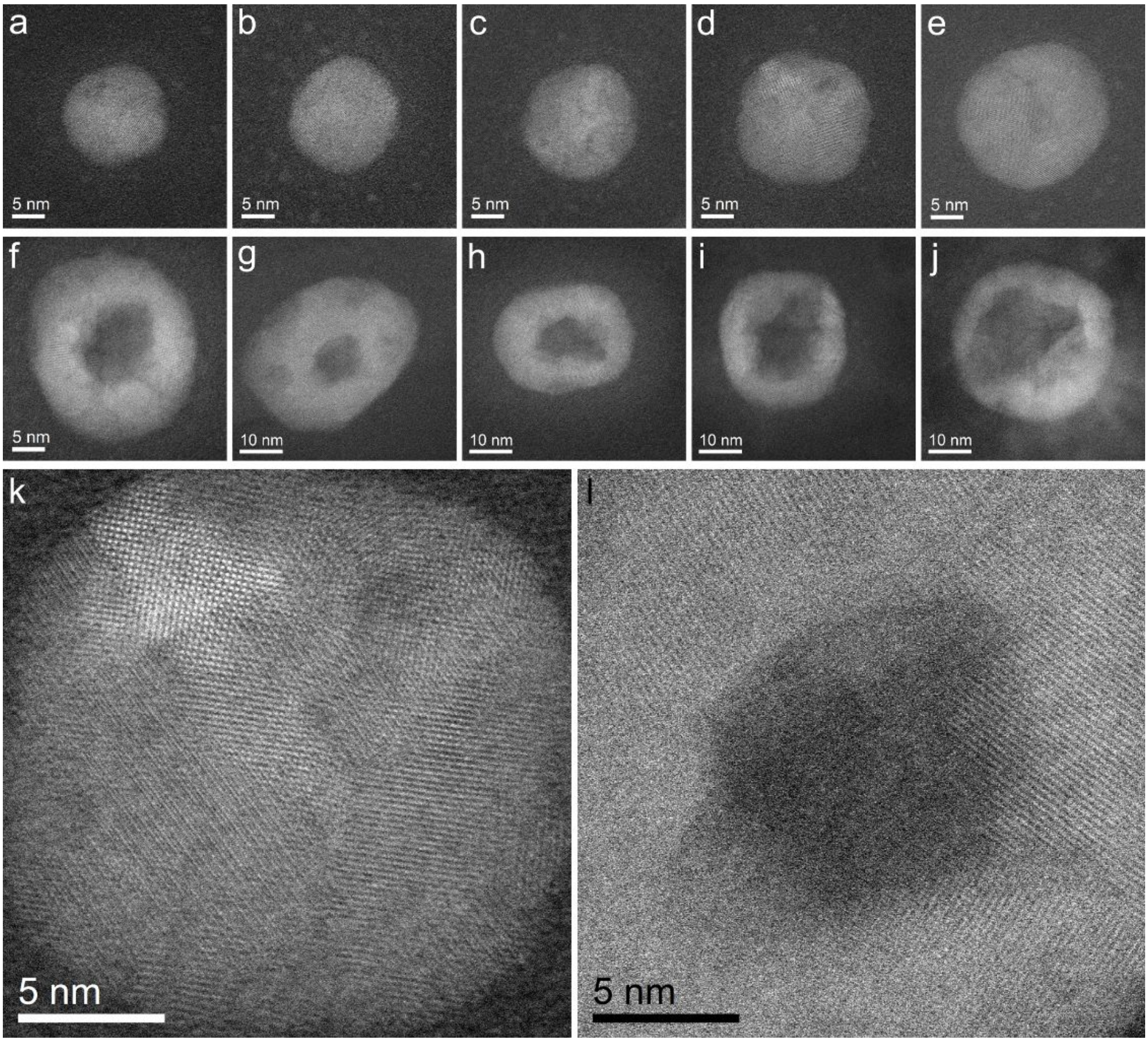

Figure S13: Graphene liquid cell STEM images of ferritin proteins in water and in 6 M urea. (a-e) High-magnification images of folded ferritin proteins in water. **(f-j)** High-magnification images of ferritin nanorings in 6 M urea. Magnified views of **(k)** the folded ferritin protein in panel **d)** and **(l)** the hollow center of the ferritin nanoring in panel **g)**. These atomic-scale images reveal that both folded and unfolded ferritins are polycrystalline.

Section S5: Preparation of apoferritin samples

Apoferritin from the equine spleen was purchased from Sigma Aldrich (CAS number: 9013314) dissolved in 50% glycerol and 0.075 M NaCl. 20 μL of the as-received apoferritin proteins at a concentration of 10 μg/mL was injected on a freshly cleaned graphene surface through the inlet port of the AFM liquid cell. The AFM measurements were conducted in tapping mode using a DLC-AFM tip.

References

1. Schitter, G.; Rost, M. J., Scanning probe microscopy at video-rate. *Materials Today* **2008,** *11*, 40-48.
2. Linder, M. C.; Kakavandi, H. R.; Miller, P.; Wirth, P. L.; Nagel, G. M., Dissociation of ferritins. *Archives of Biochemistry and Biophysics* **1989,** *269* (2), 485-496.
3. Crichton, R. R.; Declercq, J.-P., X-ray structures of ferritins and related proteins. *Biochimica et Biophysica Acta (BBA) - General Subjects* **2010,** *1800* (8), 706-718.
4. Phillips, J. C.; Braun, R.; Wang, W.; Gumbart, J.; Tajkhorshid, E.; Villa, E.; Chipot, C.; Skeel, R. D.; Kalé, L.; Schulten, K., Scalable molecular dynamics with NAMD. *Journal of Computational Chemistry* **2005,** *26* (16), 1781-1802.
5. Huang, J.; Rauscher, S.; Nawrocki, G.; Ran, T.; Feig, M.; de Groot, B. L.; Grubmüller, H.; MacKerell, A. D., CHARMM36m: an improved force field for folded and intrinsically disordered proteins. *Nature Methods* **2017,** *14* (1), 71-73.
6. Smith, L. J.; Berendsen, H. J. C.; van Gunsteren, W. F., Computer Simulation of Urea−Water Mixtures: A Test of Force Field Parameters for Use in Biomolecular Simulation. *The Journal of Physical Chemistry B* **2004,** *108* (3), 1065-1071.
7. Brenda, L.; Mary, M.; Micheal, B.; N., S. B.; Giuseppe, V.; Damien, T.; C., G. J.; M., P. I.; John, M.; Guaylord, L.; Daniel, N.; J., Q. A., Non-Covalent Functionalization of Graphene Using Self-Assembly of Alkane-Amines. *Advanced Functional Materials* **2012,** *22* (4), 717-725.
8. Textor, M.; de Jonge, N., Strategies for Preparing Graphene Liquid Cells for Transmission Electron Microscopy. *Nano Letters* **2018,** *18* (6), 3313-3321.
9. Dachraoui, W.; Keller, D.; Henninen, T. R.; Ashton, O. J.; Erni, R., Atomic Mechanisms of Nanocrystallization via Cluster-Clouds in Solution Studied by Liquid-Phase Scanning Transmission Electron Microscopy. *Nano Letters* **2021,** *21* (7), 2861-2869.
10. Dachraoui, W.; Henninen, T. R.; Keller, D.; Erni, R., Multi-step atomic mechanism of platinum nanocrystals nucleation and growth revealed by in-situ liquid cell STEM. *Scientific Reports* **2021,** *11* (1), 23965.
11. Huang, T.-W.; Liu, S.-Y.; Chuang, Y.-J.; Hsieh, H.-Y.; Tsai, C.-Y.; Wu, W.-J.; Tsai, C.-T.; Mirsaidov, U.; Matsudaira, P.; Chang, C.-S.; Tseng, F.-G.; Chen, F.-R., Dynamics of hydrogen nanobubbles in KLH protein solution studied with in situ wet-TEM. *Soft Matter* **2013,** *9* (37), 8856-8861.
12. Yuk, J. M.; Jeong, M.; Kim, S. Y.; Seo, H. K.; Kim, J.; Lee, J. Y., In situ atomic imaging of coalescence of Au nanoparticles on graphene: rotation and grain boundary migration. *Chemical Communications* **2013,** *49* (98), 11479-11481.
